# Marine Macroalgae in Topical Formulations: Bioactive Compounds, Variability, Analytical Challenges and Skin Benefits

**DOI:** 10.3390/pharmaceutics17091143

**Published:** 2025-08-31

**Authors:** Cătălina Bogdan, Mara Molnar, Elena Ines Dima, Andreea Alexandra Olteanu, Diana Antonia Safta, Mirela-Liliana Moldovan

**Affiliations:** 1 Department of Dermopharmacy and Cosmetics, Faculty of Pharmacy, “Iuliu Hațieganu” University of Medicine and Pharmacy, 12 I. Creangă St., 400010 Cluj-Napoca, Romania; catalina.bogdan@umfcluj.ro (C.B.); pop.molnar.mara@elearn.umfcluj.ro (M.M.); diana.an.safta@elearn.umfcluj.ro (D.A.S.); mmoldovan@umfcluj.ro (M.-L.M.); 2 Department of Nutrition and Medical Cosmetics, Faculty of Nursing and Health Sciences, “Iuliu Hațieganu” University of Medicine and Pharmacy, 12 I. Creangă St., 400010 Cluj-Napoca, Romania; 3 Department of Toxicology, Faculty of Pharmacy, “Carol Davila” University of Medicine and Pharmacy, 6 Traian Vuia St., 020956 Bucharest, Romania; 4 Department of Analytical Chemistry, Faculty of Pharmacy, “Carol Davila” University of Medicine and Pharmacy, 6 Traian Vuia St., 020956 Bucharest, Romania; andreea.olteanu@umfcd.ro

**Keywords:** macroalgae extracts, macroalgal bioactives, skin health, environmental influence, extraction methods, quantitative analysis, algae-based topical formulations, cosmetics, wound healing

## Abstract

Marine macroalgae, classified into three major groups, brown (Phaeophyceae), red (Rhodophyta), and green (Chlorophyta), represent a source of structurally diverse compounds relevant for topical applications. This narrative review of the peer-reviewed literature and regulatory databases targets macroalgae-derived active ingredients in cosmetic formulations and in wound-healing applications. It outlines major compound classes (polyphenols, sulfated polysaccharides, carotenoids, fatty acids, and peptides), along with their documented biological effects on skin (antioxidant, anti-inflammatory, moisturizing, photoprotective, and anti-aging activity) and regulatory/safety aspects with formulation strategies. This review also addresses the variability in compound concentrations resulting from species, environmental conditions, and seasonal factors, which impacts reproducibility and standardization. Common extraction techniques like solvent extraction, ultrasound-assisted extraction, supercritical fluid extraction, and enzyme-assisted methods are described in relation to compound class and yield. Analytical methods used for the identification and quantification of these compounds, including HPLC, GC-MS, and FTIR, are then summarized. Additionally, recent in vitro and in vivo studies evaluating the bioactivity and safety of macroalgae-derived ingredients are discussed. This review compiles relevant evidence to inform formulation strategies and ingredient evaluation in the context of marine-based topical products.

## 1. Introduction

In recent years, there has been an important increase in consumer interest in natural-based cosmetic products, especially for the valorization of natural origin ingredients. In this context, the European Green Deal represents a priority of the European Union, supporting the critical role of sustainable products and technologies in reaching climate neutrality by 2050. Achieving this objective will require the active involvement of all industrial sectors, including the cosmetic industry, which are expected to contribute significantly across their entire value chains. One of the main goals of this sustainability-driven approach is to promote the responsible and efficient use of natural resources. Among these, marine-derived ingredients, especially algae, represent a valuable source of cosmetic ingredients due to their rich content of bioactive compounds [1]. To date, numerous studies have described various applications of algal compounds, which are mainly due to their biochemical variety, sustainability and availability. Despite these advantages, only a limited number of studies have systematically presented this information. This review focuses specifically on macroalgae by presenting the extraction challenges of bioactive compounds, the environmental variability in their chemical composition, the quantitative analysis of bioactives, and their applications in skin health. To establish this context, general insights about biochemical diversity and classification that provide the framework for understanding dermatological applications of the macroalgal compounds are also presented.

Algae can be either prokaryotic or eukaryotic and display a wide range of forms and structures; structurally, they are divided into unicellular (microalgae) or multicellular (macroalgae) organisms. Over a period of 2.45 billion years, algae have evolved specific features that enable them to thrive in harsh and competitive environments, including the synthesis of a variety of biologically active molecules and secondary metabolites, with protective and adaptive functions in diverse ecosystems [2]. The biochemical diversity of algae represents a sustainable and potent resource for innovative cosmetic products [3]. Structurally, algae’s cell walls are mainly made of cellulose, hemicellulose, mucilage and pectin, while brown and red algae are rich in polysaccharides such as alginic acid, fucoidan and fucan. Photosynthetic pigments, particularly chlorophyll a and chlorophyll b, are involved in energy conversion, generating oxygen and various sugars, mainly glucose, which are further converted to starch and biomass. Another important characteristic is that algae are very resilient to extreme environmental conditions such as extreme pH levels, temperatures, osmotic stress, salinity, ultraviolet radiation, and low-oxygen conditions. In these difficult environmental conditions, algae produce metabolites such as carotenoids (lutein and zeaxanthin), vitamin E, and fatty acids like oleic acids, which protect their cellular structures. Additionally, under severe stress conditions, they synthesize secondary compounds with antimicrobial properties effective against fungi and viruses [4]. The biochemical diversity of compounds found in algae is estimated as ten times higher than that of terrestrial plants and their flavonoid profile is completely different than that of terrestrial plants [5]. The resilience of algae, together with their diverse range of bioactive compounds, demonstrates their potential in cosmetic and dermatological formulations, providing antioxidant, antibacterial, and skin-revitalizing characteristics for novel applications.

One of the earliest systematic efforts to categorize algae was made by W. H. Harvey, who played an important role in the classification of algae. Since his contributions, a variety of classifications were proposed, incorporating morphological and physiological parameters, and biochemical or molecular characteristics [6].

Beginning in the 1830s, algae were divided into main groups based on their chromatic distinctions (red, brown, and green), which correspond to different pigments found in chloroplasts, including phycobiliproteins, carotenoids, and chlorophylls. These categories reflect chromatic differences but also metabolic profiles that influence their bioactive potential. This classification led to the description of major algal classes: Phaeophyceae (brown algae), Rhodophyceae (red algae), and Chlorophyceae (green algae). Overall, it is estimated that approximately 59% of known algal species are brown, and red algae comprise about 40%, while green algae represent less than 1%. In 1989, Robert E. Lee proposed a classification system for algae based on the evolution of the chloroplast, dividing them into 4 groups and further into 15 divisions (*phyta*). The revised system, updated in 2008, reflects advancements in phycological research and taxonomy. Modern approaches also integrate morphological and molecular characteristics, including pigment composition (e.g., phycocyanin), genetic markers, fatty acid profiles, and secondary metabolite profiles, as well as biophysical parameters derived from diffraction patterns, light scattering, and fluorescence measurements [7].

## 2. Sources of Macroalgal Bioactives

Because most of the algae used in skin products come from the groups Phaeophyceae (brown algae), Chlorophyceae (green algae), Rhodophyceae (red algae), this section highlights the most important species from these groups, focusing on their specific compounds and effects on skin health. The incorporation of numerous algal species into topical formulations reflects their biochemical diversity, each addressing dermatological needs.

### 2.1. Phaeophyceae (Brown Algae) as Sources of Bioactive Compounds

*Laminaria*, one of the best-known genera, belongs to the brown algae group, which are distinguished by their high quantities of iodine, alginic acid, laminarin, potassium, and other active compounds. Oily extracts obtained from *Laminaria ochroleuca*, *Laminaria cloustoni* and *Laminaria digitata* have demonstrated protective effects against photoallergic reactions and enhance cellular metabolism. Aqueous extracts derived from *Laminaria saccharina and Laminaria hyperborea* possess anti-seborrheic properties, which are useful for oily skin. Rich in iodine, these brown algae enhance lipase activity, this effect making them valuable components in anti-cellulite cosmetic products. *Laminaria digitata* is particularly rich in proline, lysine, and glycine (constituents also found in human elastin), which makes its extracts useful to be incorporated in hair conditioners and products for improving skin elasticity. All species are characterized by a greenish brown trisected thallus. Among them, *Fucus vesiculosus* is the most frequently used species in the cosmetic industry, followed by *Fucus spiralis* and *Fucus serratus*. These species have a high content in iodine, zinc, laminarin, magnesium, manganese, and vitamin C. *Fucus vesiculosus* is often used in products for photoprotection. Moreover, this genus is also an excellent source of bioactive compounds such as fucoidans, phlorotannins and fucoxanthin, compounds with documented potential in a wide spectrum of conditions. Another species of brown algae used in cosmetic formulations is *Pylaiella*, which grows in the cold sea regions. It is extracted from the species *Pylaiella littoralis*, *Pylaiella ochotensis*, *Pylaiella flexilis*, *and Pylaiella seriata. Pylaiella* is rich in alginic acid, mannitol, laminarin, fucoidan, vitamins, polysaccharides; this extract has moisturizing and antioxidant effects, and skin renewal and skin metabolism stimulation, which will be further detailed in Section 8 [8].

Table 1 summarizes examples of cosmetic ingredients derived from brown algae, showing the industrial and formulation potential of brown algal bioactives. As can be observed, many extracts from *Laminaria* spp. are employed as humectants and skin conditioning agents, while various extracts from *Fucus* spp. have emollient, soothing, and skin-protecting functions. Additionally, genera such as *Ascophyllum nodosum*, *Hizikia fusiforme*, *Lessonia nigrescens*, and *Saccharina japonica* provide skin conditioning and protective effects in cosmetic formulations.

### 2.2. Chlorophyceae (Green Algae) as Sources of Bioactive Compounds

Species from the *Ulva* genus are among the most used green macroalgae in the cosmetic industry. These algae can have two different morphological forms: one with blistered thallus characteristic to adult thalli, and another with corrugated thalli typical of juvenile organisms. Among them, *Ulva lactuca* is the most frequently used alga in the cosmetic industry. This species contains vitamins A, B, C, E, magnesium, iron and aosaine, a protein that contains the same amino acids as human elastin (glycine, proline, lysine) and that is used in cosmetic formulations targeting aging-skin problems like wrinkles and reduced firmness [8]. *Ulva* extracts and their hydrolyzed derivatives, such as *Ulva lactuca*, *Ulva linza*, and *Ulva australis*, are officially recognized for functions like skin conditioning, emollient, and humectant activities, as shown in Table 2.

Another relevant genus for skin applications is *Cladophora* which grows in marine and freshwater environments, but it mostly grows in warm seas. The thallus of these algae is identified by its threadlike appearance and extensive branching; the cell walls are occasionally coated with calcium compounds. These algae contain siphonoxanthin, a rare carotenoid pigment. The most relevant species from this genus are *Cladophora glomerata*, *C. brasiliana*, *C. columbiana*, *C. dalmatica*, *C. fracta* and *Aegagropila linnaei.* Particularly, the extract from *C. glomerata* is widely used in cosmetic formulation. The extracts from *C. glomerata* contain high concentrations of phenolic and polyphenolic compounds that are responsible for its strong antioxidant action. Additionally, this species has demonstrated antimicrobial activity against several bacterial strains, such as *Bacillus subtilis*, *Proteus mirabilis*, and *Staphylococcus aureus* [10].

### 2.3. Rhodophyceae (Red Algae) as Sources of Bioactive Compounds

The phylum Rhodophyta, comprising more than 6000 species, exhibits a wide range of morphological characteristics, including filamentous, branched, feathered, and sheet-like thalli. In recent years, red algae have begun to be used in cosmetic applications due to their bioactive potential. Species from the genus *Ceramium* contain high ratios of amino acids, proteins, vitamins, mineral salts, and agar. Among them, *Ceramium rubrum* is frequently used in body care products. Extracts from *Ceramium rubrum* and *Ceramium kondoi* are employed in body and skin care products, as humectants, emollients, and skin conditioning agents, as summarized in Table 3. Furthermore, extracts from these algae have been shown to have antimicrobial activity. Furthermore, *Polysiphonia* sp. shows potential in cosmetic applications due to its strong antioxidant activity demonstrated by FRAP assay, which may support activity related to age-related skin problems [2,8,11].

**Figure 1:**
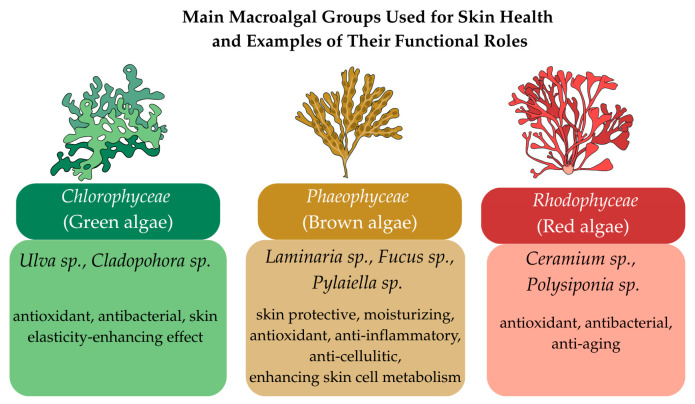
Overview of the main macroalgal groups and species used in skin health and their functional roles (image created using Canva.com, accessed on 23 May 2025).

In conclusion, green, brown, and red algae are valuable sources of bioactive compounds with a wide range of applications in skin health, which will be further explored in the following sections. Each algal group offers specific benefits, including moisturizing, antioxidant, anti-inflammatory and antibacterial effects, which justify their growing use in modern topical formulations adapted to various dermatological needs. These skin health benefits of the main algal groups are summarized in Figure 1. The following sections will provide an in-depth overview of the key active compounds, extraction and quantification methods, as well as the influence of environmental factors on the quality and efficacy of algal-derived ingredients.

## 3. Macroalgal Bioactives

Particular interest has been directed to the active compounds originating in algae, as their proven antioxidant, anti-inflammatory, moisturizing, and anti-aging effects made them relevant to the cosmetic industry, but also for other applications such as wound care. The main compoundsinclude polysaccharides, pigments (chlorophylls, carotenoids) or structures such as mycosporine-like amino acids (MAAs). Nowadays, there are many categories of skincare products formulated with different algae-derived ingredients with various roles, such as moisturizers, anti-wrinkle agents, texture-enhancing agents, and sunscreens [12].

### 3.1. Polysaccharides

Polysaccharides are large, water-soluble carbohydrates composed of long chains of monosaccharide units connected by glycosidic bonds. Polysaccharides are the most notable compounds found in algae, each type of seaweed being correlated with a specific qualitative and quantitative profile of these macromolecules: alginate, laminarin and fucoidan being predominant in Phaeophyta, agar and carrageenan in Rhodophyta, and ulvan in Chlorophyta. Within the latter, *Ulva* can contain up to 65% polysaccharides (dry weight). Other genera rich in polysaccharide are *Ascophyllum*, *Porphyra*, and *Palmaria* [12].

#### 3.1.1. Alginates

Alginates are predominant in Phaeophyta species such as *Ascophyllum*, *Durvillaea*, *Ecklonia*, *Laminaria*, *Lessonia*, *Macrocystis*, *Saccharina*, *Sargassum* and *Turbinaria*. Alginates are composed of anionic polysaccharides. There are mainly two types of uronic acid residues: β-D-mannuronic acid and α-L-guluronic acid. These exhibit anti-allergic and antimicrobial properties and are widely used in cosmetic product formulations for their film-forming, rheological, and skin-conditioning benefits [12].

#### 3.1.2. Laminarin

Laminarin is most abundantly found in brown algae, with the highest amounts recorded in species from the Laminariaceae family. Current sources of laminarin are *Laminaria digitata and japonica*, *Eisenia bicyclis*, *Saccharina latissima*, *Ecklonia kurome*, but also species with lower concentrations, such as *Fucus* or *Ascophyllum* and *Undaria*. Usually, laminarin represents around 35% of dry weight; however, this proportion may vary depending on factors like the season, the ecological growth conditions or the species itself. Laminarin has antioxidant activity, linked to an anti-inflammatory effect, and has shown antitumor potential in studies. Moreover, by degrading through irradiation, its ability to scavenge free radicals and inhibit melanin synthesis in melanoma cells is enhanced [13].

#### 3.1.3. Fucoidans

Fucoidans are sulfated polysaccharides composed primarily of fucose and other sugars such as xylose, galactose, mannose, and glucuronic acid. They also include sulfates, uronic acids, and acetyl groups. Fucoidans exhibit a variety of biological activities, including antioxidant effects through an antiradical mechanism, which vary depending on their molecular weight and sulfate content. In topical applications, fucoidans can prevent and treat skin photoaging by inhibiting UVB-induced collagenase and gelatinase activities in vitro and reducing elastase *activity* ex vivo in human skin. Additionally, they inhibit wrinkle-related enzymes, enhance collagen synthesis in human dermal fibroblasts, and provide anti-inflammatory effects by preventing extracellular matrix degradation by matrix metalloproteinases (MMP) [14].

#### 3.1.4. Ulvan

Ulvan sulfated polysaccharides represent a major component of green algae, most often being the predominant ones, varying from 8% to 29% of the algae dry weight. The main components of ulvan are rhamnose, xylose, glucose, glucuronic acid, iduronic acid, and sulfate. If divalent cations (e.g., Ca^2+^, Cu^2+^, Zn^2+^) are present in the medium, ulvan has gelling properties at pH 7.5–8.0, and remains stable up to 180 °C. Both the rheological (gelling) and biofunctional (anti-aging) properties of ulvan make it relevant as an ingredient for the formulation of cosmeceuticals. In addition, it was linked with anti-hyperlipidemic and antiherpetic effects [14].

#### 3.1.5. Agar

Agar is a gelatinous hydrocolloid structurally containing residues of two carbohydrates, agarose and agaropectin, extracted from various red algae species. Agarose accounts for 50 to 90% of agar, conferring its gelling properties. It is stable up to 250 °C and it maintains its properties even at high, near-boiling temperatures. Agar is widely used in cosmetic formulations for its thickening, emulsifying, and stabilizing properties. It is commonly incorporated into a variety of products, including moisturizers, creams, liquid soaps, cleansers, and deodorants, especially those designed for anti-aging and acne treatments [14,15].

#### 3.1.6. Carrageenan

Carrageenan is mostly present in red algae, especially in strains belonging to the order *Gigartinales*, which, for this reason, are also called carrageenophytes. These organisms generate a distinct polysaccharide known as carrageenin, which, in its natural form, is unstable and challenging to isolate. To enhance its stability, carrageenin interacts with cations, resulting in the formation of different carrageenan salts, collectively referred to as carrageenan. This compound can constitute 30% to 75% of the seaweed’s dry mass. Carrageenan exists primarily in three forms: kappa (κ), iota (ι), and lambda (λ). The kappa and iota variants are capable of forming gels, whereas the lambda type is primarily used as a thickening agent. Hence, they may be considered an alternative to cosmetic synthetic thickeners like polyethylene glycol (PEG) and carbomer [14,16].

Several of the bioactive compounds derived from macroalgae exhibit structure-dependent effects. Laminarin, a β-(1→3)-glucan with β-(1→6) branches, has a hydroxyl-rich polysaccharide backbone that provides high water-binding capacity, which confers moisturizing and barrier support properties [17]. Fucoidans, sulfated fucose-containing polysaccharides, display antioxidant, anti-inflammatory and photoprotective activities; their degree of sulfation and molecular weight determine free radical scavenging ability and inhibition of melanogenesis, thereby contributing to skin-brightening and anti-aging effects [18]. Phlorotannins, polyphenolic compounds unique to brown algae, consist of oligomeric phloroglucinol units with abundant hydroxyl groups, which confer strong radical-scavenging and tyrosinase-inhibiting activity, reducing oxidative stress, which results in anti-pigmentation and anti-aging outcomes [19].

### 3.2. Proteins, Peptides and Amino Acids

The natural moisturizing factor (NMF) has a key role in preserving the hydration, barrier function, desquamation, and flexibility of the stratum corneum. Among its key constituents, amino acids are particularly important, as they significantly contribute to maintaining the skin’s moisture balance and supporting the stratum corneum’s optimal hydration levels [16]. All macroalgae contain both essential and non-essential amino acids. Regarding the protein content, red algae have a higher content, up to 47%, compared to green algae, with a level of 9% to 26%. The lowest protein concentrations are found in brown algae, from 3% to 15%. Seaweed-derived proteins and bioactive peptides were proven to have powerful antioxidant effects, particularly the low-molecular-weight compounds. Seaweeds are rich in amino acids such as alanine, proline, arginine, serine, histidine, and tyrosine. High concentrations of arginine are found in *Palmaria* sp. and *Porphyra* sp., which acts as an NMF and is relevant in the composition of the cosmetic products. Bioactive peptides in seaweeds typically contain 3–20 amino acid residues, which contribute, according to their content and sequence, to the antioxidant and antimicrobial properties. Peptides like carnosine, glutathione, and taurine exhibit antioxidant and chelating properties, though taurine is not a “true” amino acid due to its lack of a carboxyl group. Taurine is abundant in the thalli of several red algae, such as *Ahnfeltia plicata*, *Euthora cristata*, and *Ceramium virgatum.* A relatively new peptide is PPY1, obtained from *Pyropia yezoensis* (red algae) through enzymatic hydrolysis, which has demonstrated anti-inflammatory effects by suppressing inflammatory cytokines. Additionally, peptides PYP1-5 and porphyra-334 from *Pyropia yezoensis* reduce the expression of MMP, thereby enhancing elastin and collagen production [13].

#### Mycosporine-like Amino Acids

Mycosporine-Like Amino Acids (MAAs) represent a natural means of protection against UV radiation, a relevant feature for sunscreen formulation. These small molecules with a molecular weight of less than 400 Da are found in the cell cytoplasm. Their chemical structure includes a cyclohexenone or cyclohexenimine ring connected to amino acid substitutes. The ring-substituent bonds allow MAAs to absorb a wide range of UV wavelengths, while the type of substituent influences the absorption properties [14,20].

### 3.3. Pigments

#### 3.3.1. Phycobillins

These compounds can be isolated from cytoplasm or cell stroma. They are connected by covalent bonds, which are responsible for the color, and the resulting complexes, which belong to three categories: phycoerythrin, phycocyanin and allophycocyanin. R-phycoerythrin is commonly found in Rhodophyta, but its concentration varies between species. This pigment has been widely used as a natural coloring agent in food products, cosmetics, and pharmaceutical formulations, along with other phycobiliproteins. Natural colorants are stable, and resistant to exposure to light, high temperatures, and pH changes. At present, pigments including C-phycocyanin, R-phycoerythrin, and B-phycoerythrin are incorporated into everyday coloring makeup products like eyeliners and lipsticks [14].

#### 3.3.2. Chlorophylls

Chlorophylls, the green pigments essential for photosynthesis, are located within the chloroplasts and universally present in all seaweed species, regardless of their external coloration. The visual diversity observed among seaweeds arises from varying compositions of photosynthetic pigments, with the predominant pigment determining their taxonomic classification into Chlorophyta (green algae), Rhodophyta (red algae), or Phaeophyceae (brown algae). Among these, chlorophylls are typically the most abundant pigment group. Owing to their strong antioxidant and antimutagenic effects, chlorophylls are widely utilized across food, pharmaceutical, and cosmetic industries. Their ability to absorb red and blue light while reflecting green makes them effective natural colorants. Furthermore, their documented deodorizing and antimicrobial actions, combined with their antioxidative potential, underscore their relevance as functional bioactives in cosmetic formulations [14].

#### 3.3.3. Carotenoids

Carotenoids represent a broad class of naturally occurring pigments, subdivided into carotenes—comprising α-carotene, β-carotene, and lycopene—and xanthophylls, including lutein, zeaxanthin, violaxanthin, astaxanthin, and fucoxanthin. These compounds exhibit important effects, notably antioxidant, anti-inflammatory, and anticarcinogenic actions, which support their use in natural coloring products. Their provitamin A activity is notably more stable, especially under low-oxygen conditions, compared to vitamin C. Additionally, carotenoids are capable of modulating UVA-induced gene expression, thereby providing protection against photooxidative stress, a capability particularly studied for the skin and ocular tissues. Among these, a special focus in the cosmetic industry is on astaxanthin due to its excellent safety profile and diverse biological functions, including immunomodulation, anticancer activity, and potent free radical scavenging capacity, superior to that of β-carotene. Its vibrant red pigmentation also enhances its use as a dual-purpose cosmetic additive—both functional and aesthetic. Fucoxanthin is the dominant pigment found in brown seaweeds, responsible for their characteristic olive-green to yellow-brown coloration. It is characterized by a rich pharmacological profile, including antioxidant, anti-obesity, antidiabetic, anti-inflammatory, anticancer, antimalarial, and antiangiogenic effects, making it a promising candidate for various therapeutic and nutraceutical applications [14].

### 3.4. Phenolic Compounds

Phenolic compounds are water-soluble molecules characterized by the presence of one or more hydroxyl groups attached to an aromatic hydrocarbon ring, with molecular weights ranging from 126 to 65,000 Da, according to their biotransformation through the acetate–malonate pathway. These compounds are secondary metabolites, synthesized as part of defense mechanisms against environmental stressors. According to the number of substituents, these structures can be simple phenolic compounds or polyphenols. Polyphenols include terpenoids, flavonoids, phlorotannins and bromophenols, representing a differentiation criterion from polyphenols of terrestrial origin, which mainly consist of flavonoids and gallic acid. Brown algae contain higher levels of phlorotannins, while the predominant phenolic compounds in green and red algae are bromophenols, flavonoids, phenolic acids, terpenoids and MAAs [21].

#### 3.4.1. Phlorotannins

Phlorotannins constitute an important class of polyphenolic compounds, found mainly in marine brown algae, comprising 5–12% of their dry weight. With a 1,3,5-trihydroxybenzene primary structure, they result from the polymerization of phloroglucinol units. Based on their degree of polymerization and structural variations, phlorotannins are classified into six distinct categories: phloroethols, fuhalols, fucophloroethols, fucols, eckols, and carmalols. Each class represents a unique structural form and sets of chemical properties within this group of compounds. They are able to inhibit tyrosinase activity and exhibit antidiabetic properties. The strong antioxidative, anti-allergic, and anti-inflammatory effects support the benefits these compounds can provide to cosmetic formulations. Additionally, studies using mouse skin models have demonstrated their protective effects against UV radiation. Furthermore, phlorotannins have been observed to reduce the expression of MMP-1, an enzyme involved in the breakdown of dermal collagen during skin aging [22,23].

#### 3.4.2. Phenolic Terpenoids

Phenolic terpenoids, a structurally diverse group of natural compounds, have been isolated from both brown and red macroalgae. In red algae, the most frequent types are diterpenes and sesquiterpenes, whereas in brown algae, the major representatives are meroditerpenoids, which consist of a hydroquinone core linked to a polyprenyl side chain. This class includes molecules such as plastoquinones, chromanols, and chromenes, which exhibit a range of bioactive functions. Meroditerpenoids are particularly noted for their strong antioxidant capacity and their potential to inhibit skin photoaging, but also for their good safety profile. Some derivatives have shown efficacy in minimizing UVA-induced cellular damage and mitigating photodamage in exposed skin cells, which supports their inclusion in dermatological and cosmetic formulations. Additionally, certain meroditerpenoids from brown algae have demonstrated skin-lightening or hypopigmenting effects, leading to their use as multifunctional cosmetic ingredients [14].

### 3.5. Lipids

Seaweeds generally have a low lipid content, typically of less than 5%, which is considered beneficial as lipids are highly unsaturated, consisting of ω-3 to ω-6 fatty acids. Notable polyunsaturated fatty acids (PUFAs) include α-linolenic acid (18:3*n*−3), octadecatetraenoic acid (18:4*n*−3), arachidonic acid (20:4*n*−6), and eicosapentaenoic acid (20:5*n*−3). Linoleic acid (LA) is the most prevalent PUFA in human skin, playing a crucial role in maintaining the epidermal water barrier, whose disruptions are a significant consequence of essential fatty acid deficiency. The second most prominent PUFA in the skin is arachidonic acid, which constitutes approximately 9% of the total fatty acids found in the epidermal phospholipids [23]. Additionally, seaweeds contain other lipid categories such as sterols and phospholipids. The primary sterols identified are fucosterol, isofucosterol, and clionasterol [15].

Seaweed-derived lipids have shown various beneficial activities that make them valuable in cosmeceutical formulations. For instance, E-10-oxooctadec-8-enoic acid and E-9-oxooctadec-10-enoic acid from *Gracilaria verrucosa* are recognized for their anti-inflammatory properties. Essential oils from *L. japonica*, containing compounds like tetradecanoic acid, hexadecanoic acid, (9Z,12Z)-9,12-octadecadienoic acid, and (9Z)-hexadec-9-enoic acid, exhibit significant antibacterial activity against *Staphylococcus aureus* and *Bacillus cereus*, as well as antioxidant effects through radical scavenging mechanisms (DPPH, superoxide, ABTS). Fucosterol, found in *Pelvetia siliquosa*, also enhances antioxidant defenses, this time by boosting the enzymatic activity of superoxide dismutase, catalase, and glutathione peroxidase. Studies on *Hizikia fusiformis* have revealed that fucosterol contributes to defense against UVB-related skin damage by downregulating MMPs and promoting procollagen synthesis, in addition to exhibiting significant anti-inflammatory properties. Meanwhile, the brown alga *Laminaria ochroleuca* is known for producing hydrating agents frequently incorporated into skincare formulations. Extracts from this species are particularly rich in unsaturated fatty acids, accounting for approximately 55% of their composition, and include linoleic, oleic, linolenic, and palmitoleic acids. The lipid composition of various macroalgae—such as *Ulva rigida*, *Gracilaria* spp., *Fucus vesiculosus*, and *Saccharina latissima*—has been related to their antioxidant capacity and enzyme inhibitory effects. Additionally, unsaturated lipids derived from brown seaweeds are associated with free radical scavenging properties, while the fatty acid profiles of species like *Pterocladia capillacea*, *Sargassum hornschuchii*, and *Ulva lactuca* have been explored as biomarkers for chemical stress exposure in marine environments [15,24].

### 3.6. Vitamins and Minerals

Algae are rich in vitamin A and a range of B vitamins (B1, B2, B3, B5, B7, and B12), as well as vitamins C, D, E, and K. These vitamins play essential roles in metabolic processes, often functioning as enzyme co-factors. Vitamin A is known for its antioxidant and anti-wrinkle properties, making it a popular ingredient in cosmetics aimed at reducing fine lines and hyperpigmentation. The B vitamin complex, particularly niacinamide (B3), is widely used in skincare for its ability to strengthen the skin barrier by reducing water loss and promoting the production of proteins like keratin and ceramides. Red algae and other seaweeds are excellent sources of vitamin B12, which supports hair and nail growth and has anti-aging benefits. Vitamin E, another powerful antioxidant abundant in many seaweeds, helps protect against UV damage and photoaging, especially in its non-esterified form. It is often combined with vitamin C, which regenerates oxidized vitamin E. Vitamin C, commonly sourced from red algae like *Ceramium rubrum* and *Porphyra leucosticta*, offers antioxidant, anti-inflammatory, and antimicrobial benefits. It is widely used in cosmetics to reduce pigmentation and fine lines, repair blood vessels, and promote tissue regeneration. It inhibits tyrosinase activity, reducing melanogenesis by interacting with copper ions. On the other hand, vitamin K has a protective effect on tissues, supporting wound healing and reducing bruises and scars. Together, these vitamins extracted from algae provide numerous skin benefits, making them valuable in modern cosmeceutical products [15,25].

Algae have the ability to absorb a variety of trace elements and micronutrients due to the permeability of their cell walls to small molecules. This allows them to contain elements like zinc, aluminum, magnesium, silica, copper, iodine, selenium, iron, and manganese. Additionally, they can accumulate nutrients such as calcium, sodium, phosphorus, potassium, and chlorine, but their presence and/or concentrations in algae are influenced by habitat. These minerals are crucial for metabolic processes, acting as cofactors for different catalytic enzymes [26].

**Figure 2:**
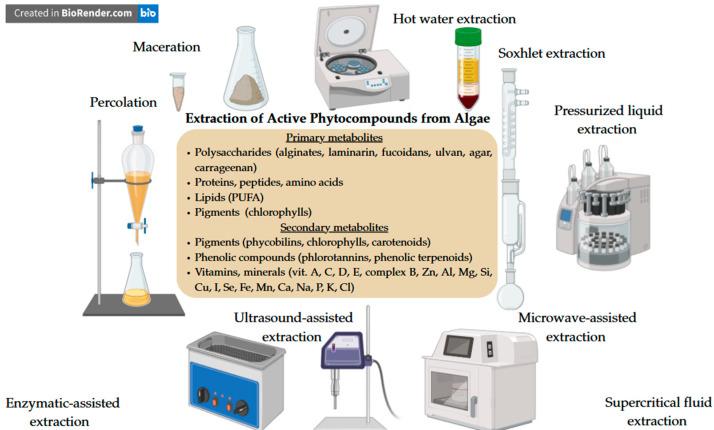
Extraction methods and active phytocompounds extracted from algae (image created using Canva.com and BioRender.com, accessed on 19 May 2025).

In conclusion, algae represent a rich and versatile source of bioactive compounds with significant potential for skin health. Their primary metabolites, such as polysaccharides, amino acids, and lipids, play essential structural and physiological roles and contribute to moisturizing, regenerating, and barrier-protective effects. On the other hand, secondary metabolites, including phenolic compounds, pigments, and certain peptides, are mainly responsible for antioxidant, anti-inflammatory, photoprotective, and anti-aging activities. The specific chemical composition corresponding to the different species of brown, red, and green algae contributes to a wide spectrum of functional ingredients suitable for topical formulations. The increasing demand for sustainable and natural topical products has an answer in algae-derived ingredients as biodegradable and biocompatible alternatives to synthetic compounds, at the same time providing undoubtable cosmetic effects. Continued exploration of their structures, mechanisms of action, and bioavailability will further expand their applications and efficacy in the cosmeceutical field. Figure 2 summarizes the main extraction methods and highlights the most important primary and secondary metabolites obtained from different algae species, as well as their cosmetic relevance.

## 4. Environmental Influence on Macroalgal Chemical Composition and Toxicity

### 4.1. Habitat

The complexity of macroalgae habitat influences the chemical composition of Chlorophyceae, Phaeophyceae and Rhodophyceae. Habitat complexity in reefs enhances herbivory, prompting macroalgae to develop defenses like calcification, toxic chemicals as secondary metabolites, or both. In contrast, algae in simpler habitats (e.g., rocky shores or lagoons) show an unclear chemical defense. In total, 6 species of green algae, 7 of brown and 18 of red were collected from different places, which were a coral reef in the Mexican Caribbean, three myxohaline areas in the Yucatán peninsula and six rocky intertidal regions (four in the Mexican Pacific, two in the Gulf of Mexico). Toxicity was interpreted in correlation with lethality caused to the goldfish *Carasius auratus auratus.* All Chlorophyceae species were highly toxic, as were four of Phaeophyceae and half of Rhodophyta (nine species). The highest toxicity was exhibited by *Caulerpa cupressoides*, *Dictyopteris delicatula*, and *Ceramium nitens*. A gradient of toxicity related to the complexity of the habitat was detected; the more toxic macroalgae extracts were found in reefs, followed by a rocky intertidal environment, and lastly in myxohaline environments. A significant relationship between habitat complexity and toxicity potency was established for Chlorophyta and Rhodophyta species [27]. Besides the secondary metabolites produced as means of defense against grazers, macroalgae may host the benthic dinoflagellate *Gambierdiscus toxicus*, which produces polyether toxins ciguatoxins, which determine ciguatera poisoning in humans, known to be mostly caused by fish consumption. Palatable macroalgae, those more likely to be harvested for food, may host *Gambierdiscus* populations if they are collected in tropical reef regions where the dinoflagellate thrives. The risk is higher for wild-harvested macroalgae in endemic ciguatera regions (Pacific Islands, Caribbean). Of special concern is the Phaeophyceae genus *Dictyota*, which hosts large populations of *G. toxicus*; it is widespread in tropical areas and produces strong chemical compounds used for defense. Similarly, the *Rodophyceae* palatable genus *Acanthopora* is consumed by various marine organisms and humans, while *Laurencia* and *Asparagopsis* are sometimes used in nutraceuticals, cosmetics, or condiments; all of them were found to host moderate levels of *G. toxicus*. Despite the relatively low dinoflagellate densities hosted by palatable turfs, as fast-growing algae, they could be linked to most of the toxin flux because of the fast resource turnover [28]. Intrinsic production of chemical compounds for defense purposes is documented for *Dictyotaceae* and *Caulerpaceae*, which are known for their high number of secondary metabolites [29].

Apart from the identification of potentially toxic compounds, the nutritional characterization of macroalgae is also relevant for oral or dermal exposure. From this point of view, Dromard et al. quantitatively assessed the concentrations of proteins, lipids, soluble and insoluble carbohydrates, as well the amount of ash, in several species of macroalgae. Although they possess great concentrations of lipids, which generate high quantities of energy, *Dictyota pulchella*, *Caulerpa cupressoides*, *Caulerpa sertularioides* and *Sargassum polyceratium* were found to have average nutritional properties because of their content in insoluble carbohydrates, which are considered low energetic compounds. On the other hand, the chemical composition of *Ceramium nitens*, *Ulva lactuca* and *Lobophora variegata*, consisting of abundant protein and soluble carbohydrates levels, provides them with high nutritional quality [29]. The specific carbon metabolism of green, brown and red algae was evaluated using a carbon stable isotope. Low values were recorded for *Rhodophyta* species, which are known for the absence of carbon-concentrating mechanisms, unlike the strong ones in *Chlorophyta* studies’ samples. The biochemical profiles varied among the groups, as red algae were rich in soluble carbohydrates, while brown algae contained higher levels of lipids and proteins, and green algae exhibited elevated concentrations of insoluble carbohydrates [30].

### 4.2. Season

It was observed that the concentration of chemical constituents of the essential oil from the brown algae *Cystoseira compressa* varied from May to August. The volatile fraction of these algae contained 104 identified compounds, that summed up 84.37–89.43% of the total oil. In the samples harvested in May, June and July, there was a high proportion of fatty acids (56, 69, and 34%, respectively), mostly palmitic acid, while the samples collected in August had a high content of alcohols, especially phytol and oleyl alcohol. Ketones had the highest concentration in July (17%), decreasing in August (13%), May (9%) and June (only 2%). Aldehydes with higher molecular weight were also assessed; tridecanal had a minimum level in June (0.37%) and a maximum level in July (0.81%), while tetradecanal was not found in June and had its maximum concentration in August (0.20%). Esters, mainly methyl arachidonate, varied from 4% in August to 10% in May and June. The contents of tri, tetra, and penta-decyl esters had a peak in June, followed by July, May and the lowest levels were recorded in August [31].

### 4.3. CO_2_ Concentration and Ocean Acidification

Ocean acidification (OA) influenced the pigment level; for example, in the red alga *Pyropia haitanensis*, it led to increased chlorophyll and phycoerythrin contents, suggesting enhanced photosynthetic pigment synthesis, possibly to optimize light capture under altered CO_2_ and pH conditions.

Increased availability of inorganic carbon enhances photosynthesis, leading to greater carbon fixation and more sugar production. The content of carbon was also modified by elevated CO_2_, as was observed in *Ulva linza*, which showed higher soluble carbohydrate accumulation. On the other hand, in *Saccharina japonica* (brown), OA induced a decrease in total soluble carbohydrates and alginate content, indicating altered energy storage and structural polysaccharide synthesis [32]. A two-week exposure of *Dictyota* spp. to OA induced an increase in macroalgal production, and the consistent carbon content was not linked to high rates of photosynthesis. Thus, *Dictyota* spp. may adjust its carbon uptake strategy to offset decreased photosynthesis, obtaining greater biomass production under acidified conditions [33]. Nevertheless, it seems that the change in chemical composition as a result of OA influence is species-specific, with some macroalgae showing little or no change, while others exhibiting strong biochemical shifts. OA can interfere with intracellular acid–base regulation, which indirectly impacts the synthesis of metabolites, proteins, and lipids. This variability is often due to the presence or absence of carbon concentrating mechanisms and the species’ tolerance to pH changes. For example, *Neosiphonia japonica* exhibited no significant change in growth under OA, indicating relative resilience [32].

The environmental pH variation, alone or combined with temperature variation, was assessed on two brown algae species, *Desmarestia anceps* and *Desmarestia menziesii*, in a 39-day experiment. Reduced pH (7.6) alone induced a significant decrease compared to ambient conditions (pH = 8) in the protein content of *D. anceps* tissues, while lipid levels were greatest under ambient conditions (1.5 °C) and lowest under elevated-temperature conditions (3.5 °C). Compared to the latter, a smaller reduction in the lipid content was caused by the joint effects of temperature and pH, proving an antagonistic influence. On *D. menziesii*, reduced pH and combined elevated temperature-reduced pH caused no significant alteration to the protein concentration, but reduced pH seemed to be beneficial as it led to an increase in the lipid level [34].

### 4.4. UV Radiation

UV radiation induces a reduction in CO_2_ fixation and effective quantum yield in macroalgae. Although the intended use of UVA enhances morphogenesis and growth in some species, increased levels decrease the maximum quantum yield and inhibit photosynthesis. UVB determines damage that takes a lot of energy for restauration, which eventually implies a decrease in the growth rate. It may also determine photoinhibition and certain parameters related to the photosynthesis process.

Regarding the specific chemical composition variation caused by UV radiation, an interesting mechanism is the increase in UV-absorbing compounds as photoprotective metabolites. Mycosporine-like amino acids (MAAs), UVA and UVB-absorbing water-soluble secondary metabolites, protect cells from radiation damage. An increase in MAAs under UVB exposure was observed in *Chondrus crispus* and *Palmaria palmata* (both red), and high levels of MAAs were noticed in different species of *Porphyra* (red). Phenolic compounds that act as antioxidants and UV screens were increased in other species, like phlorotannins in *Fucus vesiculosus*. UV exposure determines alterations in pigment content, such as the degradation of chlorophyll a and carotenoids in *Ulva pertusa*. Chlorophyll loss was detected in *Enteromorpha intestinalis* or *Laminaria japonica*, along with a drop in fucoxanthin level in the latter. The typical markers of oxidative stress were identified in certain macroalgae species as a result of UV exposure. In terms of lipid peroxidation, increased malondialdehyde (MDA) levels were detected in *Gracilaria lemaneiformis* and oxidation of membrane lipids in *Porphyra haitanensis*. Species-specific chemically qualitative profile changes in response to UV exposure were recorded, as in *Gelidium pusillum*, which also experienced DNA and pigment damage, and had weak production of MAAs compared to other red algae [32].

### 4.5. Pollution

Different mechanisms for element sequestration were recorded for brown algae. *Cystoseira*, for example, a perennial alga, uptakes the chemicals from the polluted environment unrestrictedly, which may imply poor exclusion mechanism and low adaptability to polluted marine habits. *Cystoseira* included the greatest amount of the 60 analyzed metal elements, from all types of macroalgae included in this study. On the other hand, annual algae may possess the capacity to restrict element uptake under intense pollution, as they exhibit a decrease in element concentration, possibly reflecting an active elemental exclusion mechanism. Among the annual macroalgae, green (*Cladophora*) accumulated the greatest amounts of elements, followed by brown (*Codium*) and red (*Ceramium*) [35].

The most studied pollutants are heavy metals, including cadmium, considered a chronic source of contamination. Seaweeds can chelate non-essential metals using phytochelatins, a mechanism that could result in high levels of reactive oxygen species (ROS) and reduced cellular antioxidant capacity, as well as diminished enzyme activity due to competition with essential metals. Oxidative stress triggers structural changes in amino acids, sugars, lipids, fatty acids and other compounds, as algae have primary metabolites. The exposure of *Gracilaria caudata* to cadmium (Cd^2+^ from CdSO_4_·8H_2_O) at IC_50_ (3 mg/mL) determined the variation in 20 metabolites, mostly structurally related to glyoxylate, but also ascorbate, floridoside and proline. Cd^2+^ exposure also caused the impairment of five metabolic pathways, which led to amino acid accumulation, as well as carbon biotransformation intermediate compounds and antioxidants’ responses to cadmium [36].

Chlorine dioxide (ClO_2_) water treatment in power plants and desalination plants is practiced to control biomass and biofouling. *Ulva reticulata* and *Hypnea musciformis* were exposed to ClO_2_ to assess the risk associated with this water decontamination method on different types of macroalgae. It was concluded that the levels of photosynthetic pigments chlorophyll a and carotenoids increased during the first two days of exposure and decreased afterwards. The same variation was observed for polyphenols. The increase might be a response to the oxidative stress induced by ClO_2_, which also determined an alteration in the fatty acids’ profile [37].

Another category of water pollutants is the UV organic filters in sunscreens such as oxybenzone, which was tested on the red macroalga *Gracilaria vermiculophylla* during a twenty-day exposure. The 0.1 mM concentration significantly lowered the pigment (chlorophyll a and carotenoids) concentration and the rate of photosynthesis. A 100-times lower oxybenzone concentration (0.001 mM) determined only a pigment decrease without another physiological response [38].

A current aquatic pollutant is bisphenol A, largely spread across the oceans and seas. When *Ulva rigida* (green) was exposed for 24–48 h to 40–100 mg/L bisphenol A, chloroplasts were entirely discolored. In addition, 5 mg/L bisphenol determined a growth increase by 21%, while a lower concentration of 0.1 mg/L almost doubled the algae culture within 30 days, with the stimulating effect being dose-dependent. Chlorophyll a and b levels changed within the first hours of BPA exposure and declined under BPA treatment at both high (100 mg/L) and low (0.01 mg/L) concentrations [39].

**Figure 3:**
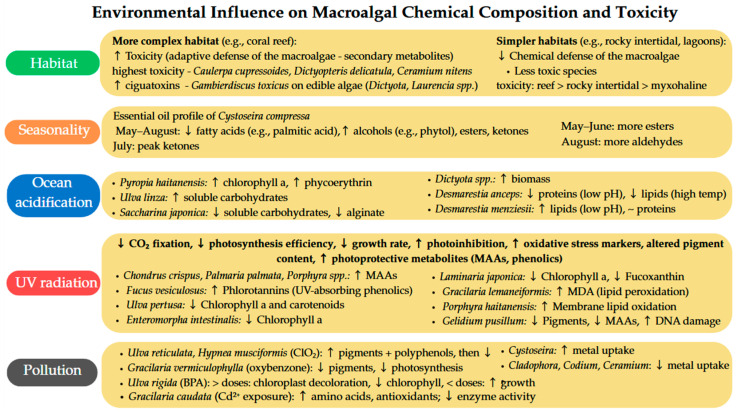
Summarization of environmental influences on macroalgal chemical composition and toxicity.

In conclusion, environmental factors—such as habitat complexity, seasonality, ocean acidification, UV radiation, and pollution—exert a significant and often species-specific influence on the chemical composition and toxicity of macroalgae. These changes not only affect their ecological interactions but also have practical implications for their safety and efficacy in human applications. Higher toxicity levels in reef-associated species, seasonal fluctuations in bioactive compounds, and alterations due to pH or pollutants emphasize the need for careful selection, monitoring, and standardization of algal biomass intended for use in topical formulations for skin health. Understanding these environmental impacts is essential to ensure the consistent quality and safety of algal-derived products. For practical purposes, we summarized these key environmental influences in Figure 3.

Environmental factors, including geographically dependent habitat complexity, seasonal variation, ocean acidification, UV radiation and pollution, have a significant and often species-specific influence on the chemical composition and toxicity of macroalgae. These changes not only affect their ecological roles but also have direct implications for their use in food, pharmaceutical and cosmetic applications. Metabolite profile variability, which includes shifts in phlorotannin levels, qualitative and quantitative differences in MAAs, lipids or polysaccharides content, can lead to inconsistent bioactivity across batches. Consequently, this may create both safety and efficacy issues, derived, for example, from fluctuant concentrations of toxic or allergenic compounds or variations in antioxidant, photoprotective or nutritional properties. Such inconsistencies pose challenges for the reproducibility of the bioactive content.

Significant variation depending on the harvest season and location was described for the protein, carbohydrate, fatty acid and mineral profiles of certain *Laminaria* species (*digitata*, *hyperborea*) or *Ascophyllum nodosum* [40]. The impact of the season on sulfated polysaccharides content and antioxidant activity was highlighted for the red algae genera *Codium* and *Osmundea* [41]. From the perspective of extraction methods, different solvents, temperatures or processing conditions can alter both the quantity and quality of the recovered metabolites. The regulatory procedure of the European Commission or FDA requires evidence for batch consistency, validated analytical methods and well-characterized toxicological profiles for considering algal ingredients for consumer use approval [42,43].

To ensure bioactive content reproducibility and consumer safety of algae and the derived products or extracts, strategies for standardized harvesting and for cultivation under controlled conditions, validated extraction methods and harmonized protocols are needed.

## 5. Regulatory and Safety Considerations of Key Compounds

The successful translation of macroalgal metabolites into cosmetic applications depends not only on their bioactivity, but also on their regulatory status and safety profile. There are two classes of compounds that are of particular interest considering their biological effects, but also their limited regulatory approval, phlorotannins and MAAs.

Phlorotannins, a particular group of polyphenols from brown algae, have exhibited potent antioxidant, anti-inflammatory and photoprotective properties [44]. Preclinical studies consistently reported favorable safety profiles, with low acute toxicity and no evidence of mutagenicity at cosmetic-relevant concentrations [42,45,46]. However, most available evidence is derived from in vitro assays or animal models, with very limited human safety data [42,47]. Importantly, phlorotannins are not yet specifically listed in the EU CosIng database, and no monographs exist under FDA or SCCS evaluations. This regulatory gap limits their routine use in European cosmetics. Moreover, the EU Cosmetics Regulation prohibits animal testing, while the REACH framework may still require animal data for complex toxicity endpoint, leading to conflicting requirements and regulatory uncertainty [42]. Although advances in alternative testing methods, such as reconstructed human epidermis assays, are promising, validation for marine extracts remains deficient [46]. Overall, phlorotannins are regarded as promising natural cosmetic ingredients, but comprehensive safety documentations and harmonized regulation and guidance are required for extended market insertion [45,47].

MAAs such as shinorine and porphyra-334 function as natural UV-absorbing compounds and have been studied as eco-friendly sunscreen filters. Toxicological assessment reports suggest excellent dermal tolerability, no genotoxicity and strong photostability [48,49]. Although some Asian regulatory authorities, such as the one in Japan, have allowed limited use of MMAs in cosmetic formulations, they are still not included in the EU list of permitted UV filters according to Regulation (EC) No. 1223/2009, nor are they FDA-approved sunscreen ingredients [49,50]. Thus, as observed for phlorotannins, validation of non-animal methods is needed, along with further human safety data, which together would permit the harmonization between the different international regulations regarding the use of MAAs in sunscreen cosmetic products.

Although a detailed discussion of toxicological evaluations, considering NOAEL values or irritation and sensitization assays, lies beyond the scope of this review, such studies are mandatory for supporting future regulatory approval of macroalgae-derived ingredients, including for cosmetic use purposes. Comprehensive reviews dedicated to toxicological milestones would enhance the quality of the present formulation and regulation-oriented perspective of the current study.

## 6. Extraction Methods of Bioactive Compounds from Macroalgae

Extraction is a crucial step in obtaining bioactive compounds from algae. After algae are collected, the biomass undergoes several processes depending on the desired compounds to be extracted. The specific process used depends on the targeted bioactive molecule. Typically, the process begins with a pre-treatment of the algal biomass such as drying the biomass either naturally or in industrial ovens at temperatures below 40 °C to prevent the degradation of sensitive bioactive compounds like proteins, vitamins, or enzymes. Alternatively, lyophilization, a vacuum-based low-temperature drying method, may be used. The dried or lyophilized biomass is then ground to a precise particle size, either for direct usage in cosmetics or to extract active components. However, to enhance cosmetic products with specific active molecules, various extraction methods are used. Extraction methods are categorized as classical or modern based on their innovation level. Classical extraction methods include percolation, maceration, Soxhlet extraction, and both solid–liquid and liquid–liquid extractions. Modern techniques, developed for greater purity and sustainability, include microwave-assisted, ultrasound-assisted, enzyme-assisted methods, and advanced processes like supercritical fluid extraction and pressurized liquid extraction. These modern approaches align with “green chemistry” principles, aiming to minimize energy use, solvent reliance, and environmental impact while enhancing efficiency [14,51].

### 6.1. Classical Extraction Methods

#### 6.1.1. Maceration

Maceration is a classic method of extraction performed on macroalgae. This technique involves soaking ground dried or fresh algal material in commonly used solvents such as methanol, ethanol or water, usually at room temperature or slightly higher temperatures, for 24–72 h. This is a gentle process that allows the solvent to penetrate the cells and dissolve active compounds: polyphenols, flavonoids, pigments and other secondary metabolites. Antioxidants and pigments can be extracted from various macroalgae. The yield and activity of the extracts depend on solvent choice, extraction time and algal species [52,53].

#### 6.1.2. Percolation

Percolation is an extraction method where a solvent is continuously passed through a column of macroalgal material, allowing for efficient extraction of bioactive compounds and larger extract volumes compared to static maceration. Its effectiveness is influenced by solvent choice and extraction conditions. Baliano et al. have utilized this technique to create bioactive methanolic algae extracts of *Padina gymnospora* for wound-healing applications [54].

#### 6.1.3. Soxhlet Extraction

Soxhlet extraction is considered a continuous solvent extraction method that selectively extracts targeted compounds from solid ones at room pressure and boiling temperature. Compared to maceration and percolation, this process uses less solvent and takes less time.

This method has multiple advantages over conventional approaches. It is faster than maceration and percolation, does not need filtration of the extract, allows for the processing of multiple samples at once and enables solvent recycling. However, there might be an increased risk of thermal degradation due to the extraction conditions: higher temperatures and long extraction time. Bhuyar et al. used this method to study the antibacterial and antioxidant properties of the red seaweed *Kappaphycus alvarezii*. The results showed that the hot water extract had a total phenolic content of 19.10 ± 0.81 mg GAE/g DW, while the ethanolic Soxhlet extract had a slightly higher content of 20.25 ± 0.03 mg GAE/g DW [55,56].

#### 6.1.4. Hot-Water Extraction

The most reliable and effective method for extracting bioactive compounds from algae, especially polysaccharides, is hot-water extraction. Since water is a harmless and non-toxic solvent that readily permeates plant tissue, hot-water extraction is the most common technique for extracting polysaccharides [57]. This technique can generate extracts that have substantial biological activity, such as antioxidant, immunomodulatory, and hypolipidemic effects, even if the yields may differ based on extraction parameters, including duration, temperature, and algae-to-water ratio. Hot-water extraction is still an excellent choice for food, medicinal, and cosmetic applications based on marine polysaccharides because of its convenience of application, low cost, and ability to maintain the integrity of sensitive compounds [58].

### 6.2. Modern Extraction Methods

#### 6.2.1. Microwave-Assisted Extraction

Microwave-assisted extraction (MAE) is an advanced technique increasingly used to recover valuable bioactive compounds from macroalgae, offering significant advantages over conventional methods. By rapidly heating the solvent and algal biomass with microwave energy, MAE breaks down cell walls and promotes the release of compounds such sodium alginate, phenolics, phlorotannins, polysaccharides, and carotenoids [59,60,61,62]. In comparison to hours-long conventional extractions, studies have demonstrated that MAE can produce higher yields and a higher total phenolic content in significantly less time (e.g., 15 min at 110 °C) while simultaneously enhancing the extracts antioxidant and enzyme-inhibiting properties [59,60,61,63]. All things considered, MAE is a fast, efficient, and environmentally friendly method to extract important bioactives from macroalgae, offering distinct advantages over conventional extraction techniques in terms of yield, quality, and environmental impact [64].

#### 6.2.2. Ultrasound-Assisted Extraction

Using ultrasonic waves to break down cell walls and promote the release of essential compounds like phenolics, polysaccharides, pigments, and proteins, ultrasound-assisted extraction (UAE) is a modern, effective method to extract bioactive components from macroalgae. Compared to traditional techniques, UAE has been demonstrated to significantly enhance the antioxidant activity and yields of active ingredients such as tannins, phenols, phlorotannins, flavonoids, and fucoxanthin [65,66,67,68,69]. Phycobiliproteins and amino acids can also be extracted with UAE, and extraction efficiency can be increased by combining it with other techniques like homogenization or maceration [66,70]. Because of the high quality and bioactivity of the extracts, the method is considered environmentally friendly, requiring less energy and solvents, and it is suitable for use in food, cosmetics, and nutraceuticals. Overall, UAE provides a fast, scalable, and sustainable method for optimizing the recovery of compounds from macroalgae [66,67,71,72].

#### 6.2.3. Enzymatic-Assisted Extraction

Enzymatic-assisted extraction (EAE) is an innovative and gentle method for isolating valuable bioactive compounds from macroalgae, using specific enzymes to break down cell walls and release target molecules such as polysaccharides and proteins. Compared to conventional chemical extraction techniques, EAE has been effectively used to extract ulvan from green macroalgae and high-molecular-weight fucoidans from brown macroalgae, often yielding larger and more native-like molecules [73,74]. Process variables including pH, temperature, and extraction time are crucial for optimizing yield and purity. The selection of enzymes, such as cellulases, alginate lyases, and proteases, can be tailored to the macroalgal species and the desired compound [73,74,75,76]. EAE is also useful for protein extraction, especially when combined with other methods like ultrasound or pulsed electric fields. This improves the extracts’ antioxidant activity and gives higher yields [76,77,78]. EAE offers a sustainable, efficient, and selective approach for obtaining high-quality bioactives from macroalgae, supporting their use in diverse biotechnological and industrial fields [78,79].

#### 6.2.4. Pressurized Liquid Extraction

Pressurized liquid extraction (PLE) is a green technology that uses high temperatures and pressures to efficiently extract bioactive compounds from macroalgae, such as fatty acids, polyphenols, carotenoids, and phycobiliproteins. By modifying the polarity and extraction temperature of different solvents, such as ethanol, water, acetone, and ethyl acetate, PLE enables the selective extraction of specific compounds. For instance, ethanol-water mixtures are useful for optimizing polyphenol and flavonoid content, producing extracts with strong antioxidant and antimicrobial activity, while PLE with ethyl acetate at high temperatures can selectively extract long-chain fatty acids including oleic, arachidonic, and eicosapentaenoic acids [75,80,81]. The yield and quality of extracted compounds are significantly impacted by the macroalgae’s drying technique—oven or freeze-drying—and the precise PLE parameters such as temperature, pressure, and solvent ratio; under ideal circumstances, some studies have found that the extraction yield can increase by up to six times for carotenoids and tocopherols [81]. Although PLE is an adaptable, fast, and eco-friendly extraction method, its efficacy is dependent on the specific parameter optimization for each macroalgal species and its desired bioactive [82,83].

#### 6.2.5. Supercritical Fluid Extraction

Supercritical fluid extraction (SFE), most commonly using supercritical CO_2_, is a green and efficient method for extracting high-value bioactive compounds from macroalgae, including fatty acids, phenolics, flavonoids and pigments such as carotenoids and chlorophylls. SFE operates at high pressures and moderate temperatures, allowing for selective extraction without the use of toxic organic solvents, preserving the integrity and bioactivity of sensitive compounds [84,85,86]. Strong antioxidant, anti-inflammatory, and antibacterial properties have been shown by SFE extracts from macroalgae, making them useful for use in food, cosmetics, nutraceuticals, and even as plant biostimulants in agriculture. Furthermore, SFE produces stable extracts with minimal batch-to-batch variation, which is crucial for industrial applications, and is considered environmentally safe and sustainable. Although SFE is quite effective for many bioactives, depending on the macroalgal species and compound polarity, other techniques, including UAE, may be more effective for extracting some polyphenols [84,85,86,87,88].

Thus, the extraction method plays a crucial role in determining the quality, stability, and bioactivity of algal-derived compounds intended for cosmetic applications. These methods are summarized in Figure 2. While classical techniques such as maceration, percolation, Soxhlet extraction and hot water extraction remain relevant due to their simplicity, modern approaches like microwave-assisted, ultrasound-assisted, enzymatic, pressurized liquid, and supercritical fluid extraction offer significant advantages. These advanced methods can align with the principles of green extraction. Ultimately, selecting the most suitable extraction method depends on the specific bioactive target, desired purity, and intended end use, with a growing emphasis on sustainability and regulatory compliance.

## 7. Quantitative Analysis of Bioactive Compounds in Macroalgae

Because algae contain a wide variety of antioxidants, pigments, polysaccharides, and other active compounds, they are becoming increasingly acknowledged as important sources of bioactive ingredients for cosmetic and pharmaceutical purposes. A variety of analytical and bioassay techniques have been developed to extract, identify, and assess the activity of chemicals originating from algae that are essential to skin health and cosmetic efficacy to maximize these advantages. The most used methods for the identification and quantification of algal compounds are chromatography, spectroscopy, colorimetry, and spectrophotometry.

Alcohols, esters, amines, and acids are among the various functional groups that can be verified to be present in algal extracts using Fourier-transform infrared (FTIR) spectroscopy [89,90]. The structural identification of the main polyphenols and phlorotannins can be performed by nuclear magnetic resonance (NMR) spectroscopy. Total phenolic content is typically determined using colorimetric assays like the Folin–Ciocâlteu test, whilst phlorotannins are determined using the 2,4-dimethoxybenzaldehyde assay [90]. Spectrophotometric techniques are also used to determine the protein content and estimate pigment [89].

Certain types of algae compounds are more suitable for specific analytical techniques, and the use of these techniques is closely related to both regulatory quality control and functional validation. To confirm the identity and purity of bioactive polysaccharides used as thickeners or moisturizers in cosmetics, polysaccharides are frequently analyzed using FTIR and NMR spectroscopy. These methods offer structural information on glycosidic linkages while methylation–GC-MS enables detailed linkage profiling. Polyphenols and phlorotannins are commonly quantified through HPLC or UHPLC coupled with MS or HRMS, as these methods effectively differentiate structurally similar oligomers and facilitate the correlation with antioxidant and anti-inflammatory bioactivities pertinent to skin protection. MAAs and pigments including carotenoids and chlorophylls are successfully analyzed using HPLC-DAD or LC-MS/MS, with spectrophotometry allowing for rapid bulk quantification for quality control purposes. This analysis of pigments directly substantiates claims regarding UV protection and photostability. The most effective method for analyzing fatty acids, sterols, and volatile metabolites is GC-MS, which guarantees precise quantification and the identification of potentially harmful or unstable substances. These customized methods not only guarantee precise characterization but also satisfy legal requirements for ingredient safety and standardization (such as ISO 16128), which call for confirmed analytical fingerprints to prove the reliability and consistency of cosmetic raw materials.

### 7.1. Liquid Chromatography

High-performance liquid chromatography (HPLC), ultra-high-performance liquid chromatography (UHPLC), and liquid chromatography–mass spectrometry (LC-MS) are widely used for analyzing polyphenols, pigments, phlorotannins, amino acids, and toxins in algae [91,92,93,94,95,96]. UHPLC with diode-array detection (DAD) and tandem mass spectrometry (MS/MS) allows for simultaneous quantification of multiple amino acids and related compounds [92,93]. Hydrophilic interaction liquid chromatography (HILIC) is effective for separating polar compounds like phlorotannins [94].

Hartman et al. developed an HILIC method for the analysis of five MAAs. The HILIC Poroshell 120 column demonstrated the best separation efficiency and peak morphologies in less than 20 min. The MAAs were eluted in the following order: asterina-330, shinorine, palythine, mycosporine-serinol, and porphyra-334. The mobile phase was water/acetonitrile with ammonium acetate at pH 6.6, which was essential to preserve selectivity. The analysis was performed on different algae species, such as *Porphyra* sp., *Porphyra* ssp., *Palmaria palmata*, *Lichina pygmaea*, *Catenella repens*, *Catenella nipae* (strain 2818), *Catenella nipae* (strain 4006), and *Catenella caespitosa* [97].

In a more recent study, a UHPLC method with a diode array detector, DAD, was developed for the determination of 11 MAAs in various algal species, such as *Gracilaria chilensis*, *Pyropia plicata*, *Porphyra columbina*, *Chondrus crispus*, and *Jania rubens*. This technique used a Phenomenex Luna Omega C18 column (1.6 µm particle size) and separated all target substances completely in less than 8 min. The mobile phase consisted of water with 0.25% formic acid and 20 mM ammonium formate (phase A) and acetonitrile (phase B) [92].

MAAs were also separated and identified by high-performance countercurrent chromatography (HPCCC) from two red macroalgae species: *Pyropia columbina* and *Gelidium corneum*. The separation was performed using a 134 mL HPCCC column with PTFE tubing and a biphasic solvent system composed of ethanol, acetonitrile, saturated ammonium sulfate solution, and water (1:0.5:1:1 *v*/*v*/*v*/*v*). This technique effectively separated highly polar chemicals with a mobile phase flow rate of 3 mL/min and a rotational speed of 1600 rpm. Among all MAAs, shinorine, porphyra-334, palythine, asterina-330, and mycosporine-serinol were successfully isolated [98].

Proteins from three different species of macroalgae, *Saccharina latissima*, *Codium* spp., and *Mastocarpus stellatus*, were extracted using aqueous and alkaline-soluble protein extraction followed by enzymatic hydrolysis with alcalase. Reversed-phase HPLC coupled with electrospray ionization quadrupole time-of-flight mass spectrometry (HPLC-ESI-QTOF/MS) was used to separate and identify 37 different peptides. A porous-shell fused-core Ascentis Express C18 column (150 × 2.1 mm, 2.7 µm particle size) and a corresponding guard column were used for the peptide separation process. A gradient elution program designed for effective peptide separation was used to create the mobile phase, which was composed of water with 0.5% formic acid (A) and methanol with 0.5% formic acid (B). A QTOF mass spectrometer equipped with Agilent Jet Stream technology was used to perform the mass spectrometry analysis in positive electrospray ionization (ESI) mode [99].

One study performed by Steevensz et al. evaluates phlorotannins in five species of brown macroalgae, including *Saccharina longicruris*, *Ascophyllum nodosum*, *Fucus vesiculosus*, *Fucus spiralis*, and *Pelvetia canaliculata*, applying a rapid and efficient ultra-high-performance liquid chromatography coupled with high-resolution mass spectrometry (UHPLC-HRMS) method using HILIC. The procedure revealed different phlorotannin profiles among species according to degrees of polymerization and allowed for the identification of a wide range of phlorotannins, including high-molecular-weight molecules up to 6000 Da [94].

Isoflavone compounds were identified and quantified in sea algae samples (*Sargassum muticum*, *Sargassum vulgare*, *Hypnea spinella*, *Porphyra* sp., *Undaria pinnatifida*, *Chondrus crispus* and *Halopytis incurvus*) using a new hyphenated technique. Samples were sonicated and extracted with supercritical CO_2_ modified by MeOH/H_2_O. Separation was carried out on a Zorbax SB-CN column using a 0.2% acetic acid–acetonitrile gradient. Detection was performed by Agilent 6460 Triple Quadrupole LC/MS in negative ESI mode [95].

A more recent study, conducted by Sardari et al. employed ultra-high-performance liquid chromatography coupled with high-resolution tandem mass spectrometry (UHPLC-HRMS/MS) to identify and characterize phlorotannins in two brown algae species: *Saccharina latissima* and *Ascophyllum nodosum*. Four phlorotannin species were identified in *A. nodosum* and one in *S. latissima* by UHPLC-HRMS, which after purification by solid phase extraction (SPE), produced purer, more concentrated fractions [100].

Yum et al. developed an analytical method for simultaneous quantification of five fatty acids in macroalgae, including myristic acid, palmitic acid, cis-palmitvaccenic acid, stearic acid, and oleic acid. The method employs SPE for sample purification and derivatization with trimethylaminoethyl (TMAE) to enhance ionization efficiency for liquid chromatography–tandem mass spectrometry (LC–MS/MS) analysis. Chromatographic separation was achieved using a CAPCELL PAK C18 MGII S3 column. The mobile phase consisted of distilled water and acetonitrile containing 5 mM ammonium acetate. The derivatized fatty acids were detected within 13.5 min using electrospray ionization in positive mode and multiple-reaction monitoring [101].

Liquid chromatography is a fundamental method for examining a variety of substances found in algae, such as MAAs, phlorotannins, amino acids, and proteins. This is essential for quality control and research on algae-based products due to its speed, sensitivity, and versatility. Quantification of these molecules also provides a direct basis for functional validation. For example, amino acids and peptides have well-established functions in hydration and barrier repair, phlorotannins exhibit antioxidant and anti-inflammatory properties pertinent to skin health, and MAAs are known to be UV-absorbing substances used in photoprotection. Therefore, the LC-based methods allow for structural correlations between algal constituents and their cosmetic bioactivities, in addition to ensuring accurate metabolite profiling.

### 7.2. Gas Chromatography

Gas chromatography, especially when coupled with mass spectrometry, is widely used to rapidly identify and profile fatty acids, esters, alcohols, carbohydrates, phytosterols, and volatile organic compounds in cosmetic macroalgae, supporting their evaluation and development as effective cosmetic ingredients.

Gu et al. used one-dimensional gas chromatography–mass spectrometry (GC–MS) and comprehensive two-dimensional gas chromatography (GC×GC) with flame ionization detection to analyze fatty acid methyl esters (FAMEs) [102]. Although these compounds were isolated from the marine benthic diatoms *Cylindrotheca closterium* and *Seminavis robusta*, the method may have applications for the assay of fatty acids extracted from macroalgae. Several columns, such as HP-5MS, DB-WAX, HP-88, and two ionic liquid stationary phases (SLB-IL 82 and SLB-IL 100) were tested. The best separation performance and lowest column bleeding were observed with the ionic liquid columns, particularly SLB-IL 82. The mobile phase consisted of helium (GC–MS) or hydrogen (GC×GC) as carrier gases, with programmed oven temperature gradients. For high-resolution profiling, GC×GC used flow modulation, whereas MS mode used electron ionization (EI). These techniques allowed for detailed characterization of the fatty acid profiles of both algae species, particularly those that contain uncommon and polyunsaturated fatty acids [102].

Shobier et al. analyzed the chemical composition and antifungal activity of extracts from six Mediterranean macroalgae: *Ulva lactuca*, *Ulva fasciata*, *Enteromorpha compressa*, *Pterocladia capillacea*, *Hypnea musciformis*, and *Padina pavonica*. The analysis was conducted using GC/MS on a Hewlett-Packard HP-5890 Series II system equipped with a capillary column (30 m × 0.25 mm, 0.25 μm) fused with phenyl polysilphenylene siloxane. The mobile phase consisted of helium as the carrier gas at 1.0 mL/min. The mass spectrometer operated in EI mode. Important bioactive substances that contribute to the extracts’ antifungal properties have been found [103].

Glycosidic linkages in unfractionated polysaccharides from five brown seaweed species, including *Macrocystis tenuifolia*, *Fucus vesiculosus*, *Alaria marginata*, *Saccharina latissima*, and *Himanthalia elongata*, were examined in a study using an improved methylation-GC-MS approach. After undergoing a three-step methylation process in heated DMSO, alcohol-insoluble residues were converted to partially methylated alditol acetates. GC was used for separation, and electron ionization mass spectrometry (EI-MS) was used for identification. The weak methanolysis-sodium borodeuteride pretreatment step made it possible to identify and quantify the uronic acid linkages in alginates. This technique allowed for detailed linkage profiling of unfractionated polysaccharides and enabled a comparison of polysaccharide composition across different seaweed species, tissues, harvest years [104].

Palaniyappa et al. developed a GC/MS method to analyze the bioactive compounds from the green seaweed *Caulerpa racemosa*. For the separation, a Shimadzu QP2020 instrument was used, which was equipped with an integrated mass spectrometer (operated in EI mode) and an SH-Rxi-5Sil-MS capillary column (30 m length, 0.25 mm internal diameter, 0.25 μm film thickness). The mobile phase consisted of helium. This technique permitted the identification of numerous bioactive metabolites such 3-hexadecene, phthalic acid, and 3,7,11,15-tetramethyl-2-hexadecen-1-ol, highlighting the chemical richness of marine algae and their potential antioxidant, antibacterial, and therapeutic applications [105].

GC-based techniques are particularly essential for the analysis of volatile and low-molecular-weight metabolites, including fatty acids, sterols, and secondary alcohols, many of which contribute to antioxidant activity, antimicrobial protection, and the maintenance of the skin lipid barrier. Accordingly, GC serves not only as a robust tool for chemical profiling but also as a way to support the functional significance of algae extracts in cosmetic compositions.

These studies suggest that liquid and gas chromatographic methods are essential for accurately identifying, quantifying, and monitoring a wide range of active compounds such as amino acids, proteins, carbohydrates, isoflavones, phlorotannins, ensuring product safety, efficacy, and compliance with regulatory standards.

To conclude, the growing interest in algae as sustainable and multifunctional sources of bioactive compounds has driven the development of advanced analytical techniques for their characterization. Liquid and gas chromatography, coupled with spectrophotometric, colorimetric, and spectroscopic methods, play a pivotal role in the identification and quantification of key metabolites such as polysaccharides, amino acids, proteins, pigments, fatty acids, and phlorotannins.

Crucially, these analytical techniques offer not only accurate chemical profiling, but also a solid basis for functional validation of bioactive compounds: accurate measurement of MAAs confirms their well-established photoprotective qualities, phlorotannin characterization supports their anti-inflammatory and antioxidant potential, and fatty acids and peptide analysis supports their contributions to skin barrier integrity and antimicrobial effectiveness.

By integrating chemical composition data with functional assays, the specific metabolites responsible for cosmetic activity can be rigorously identified. Beyond bioactivity validation, these techniques are integral to compliance with cosmetic regulatory frameworks. For example, ISO 16128 emphasizes the need for reproducibility, ingredient traceability, and natural origin verification. All of these necessitate established procedures for accurate identification and quantification. While FTIR and NMR spectroscopic fingerprints are increasingly being used to validate polysaccharide-rich extracts, chromatographic and mass spectrometric methods are essential for assuring batch-to-batch consistency of phlorotannins and pigments.

Nonetheless, challenges persist, including limited availability of reference standards for certain algal metabolites, seasonal and geographical variations in compound abundance, and the imperative for stringent inter-laboratory validation to meet regulatory requirements. Addressing these constraints is essential to ensure that algal extracts reliably demonstrate bioactivity while fulfilling the rigorous safety, efficacy, and quality standards necessary for cosmetic commercialization.

## 8. Evaluation of Biological Activities of Algae Through in Vitro and in Vivo Studies

The beneficial impact of marine algal extracts on skin health is obtained through mechanisms such as the inhibition of melanin synthesis, mitigation of oxidative stress, anti-inflammatory effects, protection against UV and anti-aging properties. Data about the activity and beneficial effects of the macroalgal-derived ingredients on acquired from in vitro, in vivo and human studies are summarized in Table 4 and Table 5.

### 8.1. Prevention of Age-Related Skin Changes

Marine algae are considered valuable components in anti-aging formulations due to their wide spectrum of demonstrated benefits. For example, *F. vesiculosus* extract has been shown to enhance skin firmness and elasticity by increasing integrin expression in dermal fibroblasts. In a clinical study involving the application of a gel containing 1% of *F. vesiculosus* extract, a significant improvement in skin elasticity was determined after a five-week treatment period [106]. In other research, the role of fucoidan-rich macroalgal extracts was assessed in dermocosmetic applications. In vitro assays demonstrated that fucoidans can significantly inhibit enzymes implicated in cutaneous aging and protein glycation. These compounds have been associated with skin-calming effects, photoprotection, and wrinkle depth reduction. Fucoidans obtained from *Ascophyllum nodosum* exhibit multiple mechanisms of action relevant to skin rejuvenation, including suppression of leukocyte elastase activity (thereby preserving elastic fiber integrity), stimulation of dermal fibroblast proliferation, enhancement of collagen synthesis, and important antioxidant activity demonstrated through DPPH and TEAC assay [13,107].

In another clinical study, extracts from *Ulva lactuca* and *Lola implexa* were incorporated into creams and serums and further applied by volunteers aged over 50 for a seven-day trial. The results indicated that serum preparations produced a superior hydration response immediately after application compared to creams. Moreover, the serum containing 10% active extract induced the most important firming effect in single-use testing. The two formulations were effective in increasing skin moisture retention and improving firmness, showing their potential in reducing visible signs of aging.

To date, numerous algae-based skincare products are currently available on the market. Among them, extracts from *Chlamydocapsa* sp. are incorporated into hydrogels and creams designed to improve the signs of cutaneous aging, to enhance the skin’s protective barrier, and to decrease transepidermal water loss (TEWL). *F. vesiculosus* extracts are included in facial treatments for their abilities to diminish dark under-eye circles and promote collagen synthesis, thereby enhancing skin elasticity. These bioactive compounds reflect the applicability of marine ingredients in modern skincare products [108,109].

*F. vesiculosus*, *Laminaria flexicaulis*, and *Ascophyllum nodosum* are the most used marine macroalgae in commercial anti-aging cosmetic formulations, owing to their increased number of bioactive compounds with skin-regenerative properties [110].

#### 8.1.1. Inhibition of Collagenase and Elastase and Implications for the Prevention of Skin Aging

MMPs, particularly collagenases, are enzymes responsible for degrading the structural matrix of the skin by targeting collagen; these changes lead to skin laxity and reduced firmness. Elastase is another proteolytic enzyme that degrades elastin, further leading to the formation of wrinkles. Therefore, the active compounds that inhibit collagenases and elastase activity are considered important and effective ingredients in anti-aging skincare products. One major factor for extrinsic skin aging is represented by prolonged UV exposure that promotes the generation of reactive oxygen species (ROS) and initiates the mitogen-activated protein kinase (MAPK) signaling cascades. This activation leads to the phosphorylation of the transcription factor activator protein-1 (AP-1) and to an increase in MMP expression. Marine polysaccharides have shown significant capacity to inhibit collagenase and elastase activity. For example, polysaccharides extracted from the brown alga *Hizikia fusiforme* demonstrated inhibitory effects on collagenase and elastase activities in cultured human dermal fibroblasts. These extracts preserved the collagen biosynthesis and downregulated MMP expression. Similarly, methanolic extracts of *Corallina pilulifera*, which are high in phenolic compounds, significantly reduced the expression of MMP-2 and MMP-9 in UV-stimulated dermal fibroblasts in a dose-dependent relationship. Additionally, phlorotannins from *Ecklonia cava*, *E. stolonifera* and *Eisenia bicyclis* have been shown to exert important MMP-1 suppression. In particular, *E. cava* phlorotannins inhibited MMP-2 and MMP-9 expression. Among these phlorotannins, compounds such as eckol, dieckol, dioxinodehydroeckol, and bieckol were identified as principal inhibitors of MMPs in human fibroblasts. Furthermore, it was observed that peptide extracts from *Poryphyra yezonensis* added to human fibroblast increased the production of elastin and collaged and decreased the expression of MMP protein [111,112].

#### 8.1.2. Hyaluronidase Inhibition Activity

Another enzyme involved in the process of aging is hyaluronidase, responsible for the degradation of hyaluronic acid. Among the algal compounds, many phlorotannin derivatives, such as fucophloroethol, fucodiphloroethol, fucotriphloroethol, 7-phloroeckol, phlorofucofuroeckol, and bieckol/dieckol, extracted from *Cystoseira nodicaulis*, have shown important hyaluronidase inhibitory activity. Additionally, other phlorotannins, like dieckol, eckol, bieckol, and phlorofucofuroeckol, obtained from *Eisenia bicyclis* and *Ecklonia kurome*, have exhibited significant hyaluronidase inhibition; of these compounds, bieckol demonstrated the most pronounced effect [111,113].

### 8.2. Antioxidant Activity

To date, numerous algal species have demonstrated significant antioxidant properties, which are important for reducing oxidative stress and protecting against cellular damage. Fucoidan isolated from *Sargassum tenerrimum* has demonstrated important antioxidant activity, effectively neutralizing DPPH and superoxide radicals. In another study, fucoidan extracted from *Costaria costata* exhibited anti-aging effects in human foreskin fibroblasts by inhibiting UVB-induced MMP-1 expression and enhancing the production of type 1 pro-collagen. Porphyran, a polysaccharide derived from *Porphyra haitanensis*, has shown significant antioxidant efficacy in vitro and in vivo. In a murine model of aging using Kumming mice, administration of porphyran led to a reduction in malondialdehyde (MDA) concentrations, an indicator of lipid peroxidation and oxidative stress. Additionally, an increased total antioxidant capacity (TAOC) and enhanced activity of critical antioxidant enzymes, including superoxide dismutase (SOD) and glutathione peroxidase (GSH-Px), were observed [113].

### 8.3. Anti-Melanogenic Activity

Since skin exposure to UV radiation stimulates melanin production in melanocytes, the application of cosmetics containing tyrosinase inhibitors represents a common approach to reduce skin hyperpigmentation. Tyrosinase is considered a key enzyme in the process of melanin synthesis, which determines the pigmentation of skin and hair color. In this regard, a study conducted by Cha and colleagues [114] investigated 43 species of marine algae to evaluate their efficacy to inhibit tyrosinase activity. The objective of the study was to evaluate the potential of marine algae as skin-whitening agents by assessing their influence on melanogenesis. Experimental models included murine melanocyte cultures and zebrafish embryos, and the study evaluated tyrosinase activity and melanin content analysis. Several species exhibited significant antimelanogenic effects, namely, *Endarachne binghamiae*, *Sargassum siliquastrum*, *Ecklonia cava*, and *Ishige okamurae Yendo*. Among these, *S. siliquastrum* and *E. cava* were particularly effective in reducing melanin levels in melanocytes, while all tested extracts induced an important inhibition of pigmentation in zebrafish larvae, an effect likely mediated through the suppression of tyrosinase activity. Other studies investigated the anti-melanogenic activity of algae-derived fucoidan. It was shown that fucoidan reduced the melanin ratio in murine melanocytes by activating the ERK pathway, leading to downregulation of melanogenesis-related proteins such as MITF and TYR. Fucoxanthin isolated from *L. japonica* has been investigated for its effects on UVB-irradiated guinea pigs and mice. The compound demonstrated effective inhibition of tyrosinase activity and melanin production under UVB exposure. Furthermore, brown algae extracts from *Sargassum polysytum* and *Padina tenuis* have been shown to significantly decrease melanin content in human epidermal melanocyte cultures. In an in vitro study, Park and collaborators evaluated the effects of *Pyropia yezoensis* on melanogenesis across multiple human cell lines, including melanocytes, dermal fibroblasts, and keratinocytes. The findings demonstrated that the extract possessed strong tyrosinase inhibitory activity and enhanced collagen synthesis by suppressing the expression of matrix metalloproteinases MMP-2 and MMP-9. Additionally, the extract enhanced the production of procollagen enzymes, suggesting that it could be used as a multifunctional cosmetic ingredient targeting skin aging and hyperpigmentation [113,115].

### 8.4. Moisturizing Activity

A good level of skin hydration plays a fundamental role in the prevention of skin aging by preserving healthy skin appearance and maintaining elasticity while reinforcing the skin barrier against environmental factors. Polysaccharides derived from algae are a sustainable and readily available resource, offering a cost-efficient solution for bioactive ingredients with moisturizing properties. In this sense, studies have shown that polysaccharides extracted from *Saccharina japonica*, a brown alga, exhibited superior moisturizing properties and water retention capacity. The polysaccharides from *S. japonica* demonstrated greater water retention capacity and hydration potential than hyaluronic acid, a valuable ingredient widely used in moisturization studies. In parallel, *Chondrus crispus*, a red alga rich in both polysaccharides and essential minerals, has been well recognized for its hydrating and moisture-enhancing properties.

In the case of green algae, extracts from *Codium tomentosum* have been shown to contribute to the regulation of water distribution within the epidermis, thereby reducing TEWL, particularly in dry environmental conditions. Additionally, DNA isolated from various marine algal sources, including *Undaria pinnatifida*, *Durvillea antarctica*, *Ascophyllum nodosum*, *Cladosiphon okamuranus*, *Pediastrum duplex*, and *Polysiphonia lanosa*, has been investigated for its capacity to enhance skin moisturization. A study carried out by Sayin and colleagues [112] assessed the antimicrobial potential of alginate extracted from *Sargassum vulgare*, evaluating its efficacy as a natural preservative in cosmetic formulations. The results indicated that this alginate extract exhibited higher antimicrobial activity against *Staphylococcus aureus*, *Pseudomonas aeruginosa*, *Candida albicans*, *Aspergillus brasiliensis*, and *Escherichia coli* compared to a commercially available herbal preservative containing glyceryl caprylate and glyceryl undecylenate. Besides this application, alginate is also used in cosmetics as a delivery system for lipophilic ingredients. This strategy provides protection of the actives and allows for their controlled release. Innovative formulations have recently included alginate-based nanofiber patches infused with spirulina extract and overlaid with polycaprolactone nanofibers. In vitro evaluations using human keratinocyte cell lines confirmed the absence of cytotoxicity for all components. Furthermore, the patches significantly improved skin hydration and demonstrated enhanced adhesive performance. In conclusion, alginates have a broad range of applications in cosmetic formulations. Their ability to maintain moisture levels in both skin and hair makes them valuable components in products such as creams, emulsions, face masks, and hair gels, where they act as humectants. In addition, alginates contribute to the physical stability of emulsions by preventing phase separation, act as effective gelling agents under acidic conditions, and are widely utilized to regulate product consistency [116].

**Table 4:** Active ingredients from macroalgae and their skin beneficial effects.

Active Ingredients from Algae	Beneficial Effects in Skin Health	Reference
*Main active ingredients from algae and their skin health benefits*		
Phlorotannins, carotenoids, photolyase, mycosporine-like amino acids	UV protection	[117]
Tocopherols, polyphenols, β-carotenoids (vitamin A), other carotenoids	Skin protection (radical scavenging and immune system stimulation)	
(Sulfated) polysaccharides, glycosaminoglycans	Skin moisturizing	
Essential and nonproteinogenic amino acids, highly unsaturated fatty acids	Skin smoothing and regeneration	
Phlorotannins, phloroglucinol and its oligomers	Skin whitening	
Low-molecular-weight fucoidans	Stimulation of collagen synthesis	
*Bioactive extracts from specific algae species and their* in vitro/in vivo *demonstrated effects*		
Alginates (*Sargassum vulgare*)	↗ antimicrobial properties (on *Pseudomonas aeruginosa*, *Staphylococcus aureus*, *Candida albicans*, *Escherichia coli*, *Aspergillus brasiliensis* strains)	[116]
Brown algae polysaccharides (*Hizikia fusiforme*)	UVB protection (protection collagen synthesis and ↘ MMPs expression in UVB-irradiated human dermal fibroblasts	[112]
Fucoidans (*Sargassum tenerrimum*, *Laminaria japonica*, *Fucus vesiculosus*)	Antioxidant activity (superoxide radical scavenging properties)	[113]
Fucoidans (*Ascophyllum nodosum*)	Anti-aging, UV protection, wrinkle reduction, antioxidant, collagen synthesis	[13]
Fucoidan (*Laminaria cichorioides*)	Beneficial effect in atopic dermatitis (inhibits production of IgE)	[118]
Laminarin	UVB protection (↘ ROS production and ↗endogenous antioxidant levels against UVB irradiation	[119]
Phlorotannins from *Cystoseira nodicaulis*, *Cystoseira tamariscifolia*, *Cystoseira usneoides Fucus spiralis*	Hyaluronidase inhibition, antioxidant activity (superoxide radical scavenging properties, lipid peroxidation inhibition)	[120]
Polysaccharides from *Saccharina japonica*	Skin moisturizing (moisture-absorption and moisture-retention abilities)	[121]
Porphyran from *Porphyra haitanensis*	Antioxidant capacity (↗ antioxidant enzyme activity-aging mice)	[122]
Porphyran from *Pyropiaa yezoensis*	Anti-inflammatory activity in LPS-stimulated macrophages	[123]
HMW ulvans	↗ hyaluronan biosynthesis in dermal fibroblasts	[124]
*Ecklonia cava*, *E. kurome*, *Ishige sinicola* extracts	Anti-acne activity *(on Cutibacterium acnes*)	[125]
*Fucus vesiculosus* extract	↗ skin elasticity	[106]
Marine complex including *Ulva lactuca*, *Lola implexa* extracts	Skin moisturizing, skin firming and tightening	[109]
*Pyropia yezoensis* extract	↗ collagen synthesis, prevents its degradation (human skin fibroblasts) ↘ melanin content (inhibits tyrosinase activity)	[115]
*Sargassum muticum*, *Osmundea pinnatifida*, *Codium tomentosum* water-based extracts	In vitro antioxidant activity (hydroxyl-radical scavenging properties)	[111]
*Undaria pinnatifida* extract (85% fucoidan) and *Fucus vesiculosus* co-extract, (60% fucoidan, 30% polyphenol)	Soothing, UV protection, wrinkle depth reduction; *Fucus vesiculosus* extract—antioxidant effect	[108]

TEWL—transepidermal water loss, MMPs—matrix metalloproteinases, LPS—lipopolysaccharide-stimulated, ↗—increasing; ↘—decreasing, HMW—high-molecular-weight.

**Table 5:** Summary of clinical studies on macroalgae-derived cosmetic formulations.

Algae Species (Type)	Formulation	Study Design (Subjects, Duration)	Outcomes	Main Cosmetic Effect	Reference
*Codium tomentosun* (green)	Moisturizing cream (5%)	7 days, 10–12 women	Hydration ↑ 2–3× vs. placebo; improved barrier	Hydration/barrier support	[126]
*Laminaria japonica* (brown)	Cream (0–15%)	Single use, 10 women	Dose-dependent hydration; 10% ↓ TEWL	Hydration/barrier support	[126]
*Rhizoclonium hieroglyphicum* (green)	Cream (0.3%)	30 subjects, 1 week	Immediate + sustained hydration; superior to glycerin	Hydration	[126]
*Sargassum hornieri* (brown, fucoidan)	Lotion (1%)	3 weeks, forearm study	↓ TEWL; ↑ hydration; restored barrier	Barrier function/hydration	[127]
*Fucus vesiculosus* + *Ulva lactuca* (brown + green) +ectoin	Serum mist (split-face)	28 days, 33 adults	+17% hydration; pH normalized	Hydration/wrinkle reduction	[128]
Brown algal alginate + calcium	Sheet mask	Single use	↑ hydration, pH regulation; ↓ wrinkles, ↑ smoothness; maintained microbiome diversity; ↓ *Corinebacterium*	Hydration/microbiome/anti-wrinkle	[129]
*Fucus vesiculosus* (brown)	Cream (1%)	5 weeks, 10 women	↑ elasticity, 7–8% ↓ skin thickness	Firming/anti-aging	[126]
*Undaria pinnatifida* + *Fucus vesiculosus* (brown)	Gel (0.3%)	UVB test, 25 subjects	↓ UV erythema, ↓ TEWL	Photoprotection/anti-aging	[126]
*Fucus vesiculosus* (brown, fucoidan/polyphenols)	Gel (0.3%)	60 days, 30 subjects	↑ brightness, ↓ wrinkles, ↓ age spots	Brightening/anti-aging	[126]
*Porphyra umbilicalis* (red, MAAs)	Cream (0.005%) vs. suncreen	4 weeks, 20 women	UVA protection; ↑ firmness, ↑ smoothness	Photoprotection/anti-aging	[130]
*Ascophyllum nodosum* (brown) + herbal extract	Botanical regiment	18 weeks, 56 women; 1 year extension	Comparable to hydroquinone/tretinoin; no rebound pigmentation	Anti-pigmentation	[126]
*Laminaria digitata* + *Gelidium cartilagineum* + *Pelvetia canaliculata* (brown + red)	Anti-cellulite cream	4 weeks, 90 women	↓ thigh/waist/hip circumference; ↑ firmness	Slimming/firming	[126]
*Fucus vesiculosus* + *Furcellaria lumbricalis* (brown + red)	Anti-cellulite cream	12 weeks, 35 women	↓ cellulite grade; ↓ adipose thickness	Anti-cellulite/firming	[126]
*Laminaria digitata* (brown, oligosaccharide-zinc complex)	Gel	8 weeks (acne vulgaris	↓ lesion severity; ↓ sebum; ↓ P.acnes	Anti-acne	[131]

In the studies reviewed in Table 4, some algae-derived ingredients were tested as whole extracts, while others were evaluated as isolated compounds, making a direct comparison of their efficacy challenging.

In vitro and in vivo preclinical studies provide consistent evidence for supporting the antioxidant, anti-inflammatory and skin-protective properties of macroalgal extracts and ingredients. These findings request further validation in human studies, which are compiled in Table 5.

The wide spectrum of biological effects exhibited by macroalgal extracts, mainly antioxidant, anti-inflammatory, anti-melanogenic and anti-aging activities, together with the enhanced skin moisturization effect, justifies their increasing use in topical formulations. From a practical perspective, marine macroalgae represent not only a sustainable and renewable resource, but also high-potential compounds for the development of multifunctional cosmetics intended to prevent skin aging, improve hydration and support skin health. This corresponds to the current industry trends that promote multifunctional, bio-based formulations with multiple skin benefits within a single product.

While these multifunctional benefits support the growing use of macroalgal extracts in topical products, their market adoption remains challenging. As observed in Table 1, Table 2 and Table 3, in regulatory databases such as COSING, macroalgal ingredients are classified as “skin conditioning,” reflecting the broad accepted effects focused on improvements in the skin’s overall condition, as outlined by Cosmetic Products Regulation (EC) No. 1223/2009 and Regulation (EU) No. 655/2013. While scientific studies report more specific effects, such as antioxidant activity, wrinkle reduction or photoprotection, these require rigorous validation through standardized assays and clinical trials to meet regulatory standards. Thus, the general classification in COSING contrasts with targeted bioactivities described in the research studied. Therefore, future research should focus on the validation of these claims through well-designed and well-conducted methodologies and their acceptance by regulatory authorities.

### 8.5. Skin Regeneration Activity

Another important application of marine algal extracts lies in their demonstrated effects in wound care. Table 6 highlights some of topical systems loading macroalgae-derived compounds, such as ulvan, laminarin and fucoidan, with demonstrated biological activity in skin regeneration. These compounds have pro-healing properties by modulating different phases of the wound-healing process, and by promoting wound contraction, collagen synthesis and reducing the inflammatory response. Although these compounds exhibit beneficial effects per se, their efficacy is often significantly enhanced when combined with antibacterial agents or antibiotics and incorporated into advanced delivery systems, such as hydrogels, wound patches or nanofibrous scaffolds. For example, fucoidan has been successfully incorporated into a gelatin/oxidized carboxymethyl cellulose hydrogel, resulting in accelerated re-epithelialization and reduced inflammatory response demonstrated in in vivo models [132]. Ulvan incorporated into nanofibrous patches or silver-loaded hydrogels has shown significant contributions to wound contraction, decreased inflammation at wound sites and improved vascularization, leading to an accelerated healing process [133,134]. In the case of laminarin, its combination with silver nanoparticles or 3D graphene foams promoted antibacterial activity and enhanced cell migration, thus showing the tissue regenerative potential [135].

In conclusion, the current research direction has evolved from simple phytocompound isolation and development of basic formulations to the incorporation of macroalgal-derived actives into performant, functionalized wound care formulations with controlled release and high biocompatibility.

## 9. Formulation Strategies and Challenges

For their use in topical products, algae are typically processed as algae extracts or as powders. It is essential—as previously discussed—to ensure a high quality and safety of these products, and thus, for these to be standardized in active compounds and well characterized. An additional critical aspect is the concentration selected for incorporation into the final formulation. As algae extracts show high and wide-ranged activity, their reported content in cosmetic formulations usually does not exceed 10% by weight and the average is below 5% by weight [151]. However, at present, there are no specific regulations establishing the maximum permissible levels of algae-derived compounds in skincare and cosmetic products, which adds complexity to the regulatory framework. Moreover, the absence of strict criteria for evaluating the safety of algal production further complicates their integration into the international market [152]. Despite their great potential, several challenges arise in the formulation of algal bioactives. Many compounds—such as polyphenols, polysaccharides, and carotenoids—exhibit low chemical stability, being especially vulnerable to oxygen, light, high temperatures, enzymatic reactions, heavy metal exposure, and extended periods of storage [153,154,155].

An adequate delivery system allows researchers to improve the performance and stability of the ingredients of interest [156]. The choice of formulation strategy largely depends on the type of algae extract, its solubility and the concentration to be incorporated, as well as the intended characteristics of the topical product. It has been shown that emulsions and encapsulation of the algae extracts are one of the most used strategies that allow for the extension of the stability of the compounds of interest [153]. The primary strategy to protect functional ingredients and enhance their performance upon skin contact is encapsulation. Commonly employed delivery systems in cosmetics include vesicular carriers (liposomes, silicone vesicles, niosomes, transfersomes, and other systems), emulsion-based systems (microemulsions, nanoemulsions, multiple emulsions, liquid crystals, and Pickering emulsions), and particulate carriers (nano- and microparticles, porous polymeric systems, cyclodextrin complexes) [151]. These carriers can subsequently be incorporated into emulsions, suspensions, or other topical formulations. An effective delivery system not only improves the stability and bioavailability of sensitive compounds but enhances their overall performance [151]. For example, Lourenço-Lopes compared the stability of fucoxanthin as a free molecule, in an emulsion, or encapsulated, and reported that fucoxanthin encapsulation is considered one of the best approaches, followed by its emulsion [153]. Importantly, formulations must rely on safe, eco-friendly technologies and account for scalability in manufacturing [151].

To highlight their practical importance, Table 7 summarizes some commercially available topical products enriched with various algae species, indicating their product types and claimed effects, to highlight their practical applications.

According to Table 7, algae were incorporated in multiple types of cosmetic products, including creams, serums, shampoos, and solid soaps, illustrating the versatility of macroalgae extracts in skincare, although they are most commonly formulated in creams (emulsions). A wide range of algae species are used in commercially available products, from red algae (e.g., *Jania rubens*) to brown algae (e.g., *Fucus vesiculosus*, *Fucus serratus*, *Laminaria japonica*, *Laminaria digitata*, *Laminaria cloustoni*) and green algae (e.g., *Ulva lactuca*), highlighting the diversity of bioactive compounds that can be harnessed for cosmetic purposes. The claimed effects of these products encompass moisturizing, antioxidant activity, skin barrier repair, anti-aging, UV protection, and skin regeneration, reflecting the multifunctional potential of algae-derived compounds. Notably, some products combine multiple algae species, potentially leveraging synergistic effects to enhance their benefits in skin health.

Besides the examples from Table 7, a noteworthy case is also represented by the large number of skincare products based on algae extracts from the cosmetic brand Algologie^®^ (France). Its formulations are centered around the patented Algo4^®^ complex, which combines extracts from brown macroalgae and red microalgae (*Undaria pinnatifida*, *Porphyridium cruentum*) with coastal plants extracts, providing moisturizing, protection, and anti-aging effects. The whole product portfolio covers a wide range of applications, including moisturizing, purifying, energizing, and anti-aging effects. The product range includes lotions, creams, gel-creams, foams, serums, demonstrating the feasibility of incorporating multiple marine-derived actives into various modern cosmetic formulations [164]. Other examples are from the Thalgo^®^ (France) skincare brand specializing in using marine ingredients and advanced technologies to create products aimed at revitalizing, moisturizing, and protecting the skin. A representative product from Thalgo^®^’s portfolio is Micronised Marine Algae, a powder product designed for steam baths or at-home thalassotherapy that helps to remineralize and revitalize the skin. The active ingredients include micronized marine algae powders from species such as *Fucus vesiculosus*, *Laminaria digitata*, and *Lithothamnion calcareum*. Thalgo^®^ also includes algae in a wide range of skincare products, including creams, serums, masks, cleansing gels, all formulated with marine ingredients to address diverse skin needs [165].

This diversity illustrates the versatility of marine-derived extracts and their feasibility for incorporation into different types of topical formulations, as well as their use as powders. This overview demonstrates the practical application of algae extracts in real-world topical formulations and underscores their growing relevance in modern skincare.

## 10. Conclusions

This review represents a synthesis of marine macroalgae-derived compounds relevant to skin health. The novelty of this work lies in the broad discussion from taxonomic, ecological, and biochemical data to modern extraction techniques and quantitative analysis of active compounds. Marine macroalgae, brown, red, and green, represent a promising and sustainable source of structurally diverse bioactive compounds with significant potential in topical dermatological and cosmetic formulations. Their metabolites, including polysaccharides, polyphenols, peptides, lipids, pigments, and vitamins, have demonstrated multifunctional biological activities such as antioxidant, anti-inflammatory, moisturizing, photoprotective. Nevertheless, the chemical composition of macroalgae is subject to variability influenced by taxonomic differences, environmental conditions, and seasonal dynamics, posing challenges for reproducibility and standardization in product development. Recent advancements in extraction methods (e.g., ultrasound-assisted extraction, supercritical fluid extraction) and analytical techniques (HPLC, GC-MS and FTIR) have improved the efficiency and selectivity of compound isolation and quantification. In addition to the description of isolated bioactives, this review includes evidence from in vitro and in vivo studies that demonstrate the biological activities in skin applications, considering the efficacy and dermal safety, particularly in the context of wound-healing and anti-aging interventions. Moreover, this review highlighted the diversity of commercially available products loading algae, demonstrating their practical application and huge interest. It also outlined the formulation strategies employed in topical products, noting that while incorporation into emulsions remains the most common approach, encapsulation into modern delivery systems represents a key advancement for enhancing their performance. These findings prove the usefulness of integrating macroalgae-derived compounds as effective and biocompatible ingredients in modern cosmeceutical formulations. The present review aimed to provide a theoretical basis for formulators and scientists in developing safe, effective and multifunctional skin health products within the current sustainability trends.

## Figures and Tables

**Table 1:** Examples of cosmetic ingredients obtained from brown algae [9].

INCI Name/Description	Function
Fucoidan (Sulfated polysaccharide)	Antioxidant Skin conditioning
Fucoxanthin (Carotenoid from *Sargassum siliquastrum*)	Skin conditioning
Sargachromanol F (Chromene from *Sargassum siliquastrum*)	Skin conditioning
Sargachromanol D (Chromene from *Sargassum siliquastrum*)	Skin conditioning
Sargachromanol E (Chromene from *Sargassum siliquastrum*)	Skin conditioning
*Sargassum filipendula* extract	Skin protecting
*Sargassum fusiforme* extract	Skin protecting
*Pylaiella littoralis* extract (Extract of the whole plant)	Skin protecting
*Laminaria longissima* extract	Humectant
*Laminaria digitata* extract	Skin protecting
*Laminaria diabolica* extract	Humectant Skin conditioning
*Laminaria hyperborea* extract	Skin protecting
*Laminaria ochroleuca* extract	Skin conditioning
*Laminaria cloustoni* extract (Kelp extract)	Skin protecting
*Laminaria saccharina* extract (Extract of the thallus of *Laminaria saccharina*)	Skin protecting
*Laminaria japonica* extract (Extract of the Japan kelp/seaweed, *Laminaria japonica*)	Skin protecting
Deaminated *Laminaria japonica* extract (Extract of *Laminaria japonica* extract enzymatically deaminated)	Skin conditioning
*Laminaria digitata* powder (Powder from the dried and ground thallus of *Laminaria digitata*)	Skin conditioning
*Laminaria japonica* powder (Powder from the dried and ground alga of *Laminaria japonica*)	Skin conditioning
*Laminaria digitata* water (Aqueous solution of the steam distillate obtained from *Laminaria digitata*)	Skin protecting Skin conditioning
*Fucus vesiculosus* (The algae *Fucus vesiculosus*, Fucaceae)	Skin conditioning
*Fucus spiralis* extract	Skin conditioning—emollient
*Fucus serratus* extract	Skin protecting
*Fucus vesiculosus* extract (Extract of the dried thallus of the Bladderwrack, *Fucus vesiculosus* L., Fucaceae)	Skin conditioning—emollient Smoothing Soothing
*Fucus vesiculosus* powder (Powder obtained from Bladderwrack, *Fucus vesiculosus* L., Fucaceae)	Skin conditioning
*Fucus crispus* thalle extract (Extract from the dried thallus of *Fucus crispus*, Fucaceae)	Perfuming
Hydrolyzed *Fucus vesiculosus* extract (Hydrolyzed iodinated extract from the Bladderwrack, *Fucus vesiculosus* L., Fucaceae)	Skin conditioning Smoothing Soothing
*Fucus crispus* thalle oil/(Essential oil from the dried thallus of *Fucus crispus*, Fucaceae)	Perfuming
*Fucus vesiculosus* thale oil (Essential oil obtained from the dried thallus of the Bladderwrack, *Fucus vesiculosus* L., Fucaceae)	Perfuming
Hydrolyzed *Fucus vesiculosus* protein(Hydrolysate of the protein obtained from the Bladderwrack, *Fucus vesiculosus* L., Furaceae, derived by acid, enzyme or other methods of hydrolysis)	Skin conditioning
Macrocystis pyrifera extract	Skin conditioning
Macrocystis pyrifera juice(Juice expressed from the whole plant)	Skin conditioning
*Macrocystis pyrifera* protein (Protein from the Giant Kelp, *Macrocystis pyrifera* L., Lessoniaceae)	Skin conditioning
*Macrocystis pyrifera*	Viscosity controlling
Kelp sulfated oligosaccharides (Obtained from the seaweed kelp (*Macrocystis pyrifera*, Lessoniaceae) hydrolysate)	Hair conditioning
*Sargassum horneri* powder (Powder from the dried and ground *Sargassum horneri*)	Humectant Skin conditioning—humectant
*Sargassum fulvellum* extract	Skin conditioning
*Sargassum macrocarpum* extract	Skin conditioning—emollient
*Sargassum aquifolium* extract	Skin conditioning—miscellaneous
*Sargassum ilicifolium* extract	Skin conditioning—miscellaneous
*Sargassum oligocystum* extract	Skin conditioning—humectant
*Sargassum hemiphyllum* extract	Antioxidant Skin protecting Skin conditioning—miscellaneous
*Sargassum yezoense* extract	Antimicrobial
*Sargassum serratifolium* extract	Antioxidant
*Sargassum vulgare* extract	Skin conditioning
*Sargassum muticum* extract	Skin protecting
*Sargassum glaucescens* extract	Antioxidant
*Sargassum thunbergii* extract	Antimicrobial
*Sargassum siliquastrum* extract	Skin conditioning
*Sargassum fusiforme* extract	Skin protecting
*Sargassum pacificum* thallus extract	Skin conditioning—emollient
*Sargassum fluitans*/Natans extract	Emulsion stabilizing
Hydrolyzed *Sargassum horneri* extract/Hydrolysate of *Sargassum horneri* extract derived by acid, enzyme, or other methods of hydrolysis)	Skin conditioning—miscellaneous
Hydrolyzed *Sargassum thunbergii* extract (Hydrolysate of *Sargassum thunbergii extract* derived by acid, enzyme or other methods of hydrolysis)	Antimicrobial Hair conditioning Humectant Skin protecting Skin conditioning—humectant
*Ecklonia/Laminaria* extract (Extract of mixture of *Ecklonia* and *Laminaria* algae)	Skin conditioning
*Ascophyllum nodosum*/*Fucus vesiculosus* extract	Humectant Skin conditioning
*Ascophyllum nodosum*/*Fucus vesiculosus*/*Laminaria cloustoni*/*Laminaria digitata* extract	Skin conditioning—emollient
*Ascophyllum nodosum*/*Fucus vesiculosus*/*Hizikia fusiforme*/*Kjellmaniella gyrata*/*Lessonia nigrescens*/*Saccharina angustata*/*Saccharina japonica*/*Undaria pinnatifida* extract	Skin conditioning

**Table 2:** Examples of cosmetic ingredients obtained from green algae [9].

INCI Name/Description	Function
*Cladophora wrightiana* extract	Skin conditioning
*Ulva australis* extract	Skin conditioning—emollient
*Ulva rigida* extract	Skin conditioning
*Ulva linza* extract	Skin conditioning—emollient
*Ulva lactuca* extract	Skin conditioning Skin protecting
*Ulva ramulosa* callus culture extract	Skin conditioning
*Ulva lactuca* powder (Powder obtained from dried and ground *Ulva lactuca*, Cyperaceae)	Absorbent Binding Viscosity controlling
Hydrolyzed *Ulva pertusa* extract (Hydrolysate of *Ulva pertusa* extract derived by acid, enzyme or other methods of hydrolysis)	Skin conditioning
Hydrolyzed *Ulva lactuca* extract (Hydrolysate of *Ulva lactuca* extract derived by acid, enzyme or other methods of hydrolysis)	Skin conditioning
Hydrolyzed *Ulva linza* leaf extract (Hydrolysate of *Ulva linza* extract derived by acid, enzyme or other methods of hydrolysis)	Skin conditioning—emollient Skin conditioning—humectant

**Table 3:** Examples of cosmetic ingredients obtained from red algae [9].

INCI Name/Description	Function
*Ceramium kondoi* extract	Humectant Skin conditioning
*Ceramium rubrum* extract	Skin conditioning—emollient Humectant Skin conditioning
*Polysiphonia brodiei* extract	Skin conditioning
*Polysiphonia lanosa* extract	Skin conditioning
*Polysiphonia elongata* extract	Humectant Skin conditioning
*Polysiphonia morrowii* extract	Antioxidant

**Table 6:** Topical systems loading macroalgae-derived compounds and their effects in wound healing.

Phytocompound-Based Topical Systems	Biological Activity Beneficial in Wound Healing	Reference
*Ulvan*		
Nanofibrous patches (ulvan, polyethylene oxide)	In vivo study: anti-inflammatory, antioxidant effects, ↘ inflammation, restoring biophysical parameters of skin	[133]
Wet-spun fibers, 3D printed hydrogel	In vitro cell studies: higher cell viability than alginate and chitosan in biocompatibility tests	[136]
Polycaprolactone-ulvan fibrous composite mats	In vitro cell studies-NIH3T3 fibroblasts: ↗ cellular proliferation, ↗ expression α-SMA and MMP-9 genes	[137]
Ulvan/gelatin-based nanofibrous patches	In vivo study with burn wound mouse model: faster wound contraction during early stages of healing, ↘ inflammation, uniform wound closure	[138]
Chitosan-ulvan hydrogels with cellulose nanocrystals and epidermal growth factor	In vitro cytocompatibility studies, in vivo wound-healing study on mice: epithelial regeneration and collagen deposition	[139]
Ulvan-based hydrogel matrix loaded with silver nanoparticles and human umbilical cord mesenchymal stem cell lyophilized powder	In vitro antibacterial activity, cytocompatibility, ↗ cell proliferation and cell migration, In vivo study with type II diabetes mellitus mouse model, ↗ wound-healing effect	[140]
Ulvan/Silver nanoparticle hydrogel films	In vitro antimicrobial activity, In vivo study with Wistar rats with second-degree burns accelerated wound healing, modulating inflammation, ↗ re-epithelialization, ↗ vascularization	[134]
Crosslinked ulvan/Chitosan complex films	In vitro studies: biocompatibility, scratch assay—↗ HaCaT cell migration and proliferation In vivo study with Sprague Dawley rats: regeneration of dermis, collagen production	[141]
*Laminarin*		
*Cystoseira barbata* laminaran based cream	In vitro antibacterial and antioxidant activity In vivo study with Wistar rats: ↗ collagen deposition, ↗ fibroblast and vascular density	[142]
Hydrogel patch of methacrylated laminarin loaded with ciprofloxacin	In vitro antibacterial activity, biocompatibility human dermal fibroblasts	[143]
3D graphene foam/laminarin hydrogel composite scaffold	In vitro: biocompatibility ↗ cell migration	[144]
Dialdehyde-modified laminarin- silver nanoparticles	In vitro antibacterial activity and inhibition of biofilm formation Cell viability and scratch assay: ↗ migration of human HaCaT	[135]
*Fucoidan*		
Gelatine/fucoidan nanogel-coated silver nanoparticles	In vitro antibacterial activity and inhibition of biofilm formation Cell viability and scratch assay: ↗cell regeneration	[145]
Fucoidan-loaded gelatin/oxidized carboxymethyl cellulose hydrogel	In vitro: cytocompatibility—RAW 264.7 macrophages, human dermal fibroblasts; protection against oxidative stress, ↘ NO production in LPS-stimulated RAW 264.7 macrophages, ↗ collagen synthesis and cell migration in HDF cells In vivo wound-healing model- mice: ↗ full-thickness wound healing	[132]
Fucoidan loaded PVA/Dextran blend electrospun nanofibers	In vivo studies-Sprague Dawley rats: ↗ wound-healing rate: ↘inflammatory response through antioxidant effects, ↗ epidermal regeneration and collagen deposition	[146]
Fucoidan-coated cotton dressing loaded with silver nanoparticles	In vitro antibacterial activity, cytotoxicity fibroblast cell line, scratch assay In vivo infected wound mouse model: inhibition of bacterial infection, tissue proliferation and collagen deposition	[147]
Fucoidan-loaded neutrophil membrane-coated nanoparticles	In vitro antibacterial activity, intracellular uptake ability of RAW264.7 cells In vivo MRSA-infected trauma mouse mode: long term antibacterial effect, accumulation in the infection site, ↗ infected wound closure	[148]
Moxifloxacin chitosan/fucoidan nanoparticle-loaded pullulan microneedle patch	In vitro antibacterial activity Ex vivo permeation study, cytotoxicity In vivo biocompatibility, BALB/c mice infectious wound model: accelerated wound healing, rapid wound closure	[149]
Fucoidan confined gold nanoparticles hydrogel	In vitro antibacterial activity, blood compatibility, cytocompatibility, anti-inflammatory assay on LPS-induced Raw 264.7 macrophage model, *S. aureus*-infected full-thickness wound-healing test: adherence to wound site, ↗ tissue regeneration, ↘ bacterial growth, ↘ inflammatory responses	[150]

↗—increasing; ↘—decreasing.

**Table 7:** Commercially available topical products loaded with algae (non-exhaustive list).

Algae Species	Product Type	Claimed Effects	Ref.
*Jania rubens* extract	Serum	Moisturizing, smoothing, antioxidant, plumping, detoxing	[157]
*Undaria pinnatifida*, *Ecklonia cava*, *Laminaria japonica*, *Hizikia fusiforme*, *Porphyra yezoensis*, *Laminaria digitata*, *Laminaria cloustoni*, *Enteromorpha compressa*, *Codium tomentosum*, *Codium fragile*, *Agarum cribosum*, *Ulva lactuca* extracts	Cream	Moisturizing, smoothing, skin barrier-repairing, protecting, nourishing	[158]
*Ascophyllum nodosum*, *Fucus vesiculosus*, *Crithmum maritimum*, *Corallina officinalis* extracts	Cream	Moisturizing, regenerating	[159]
*Prasinococcus capsulatus* exopolysaccharides	Cream	Protecting, hydrating, anti-aging	[160]
*Fucus serratus* extract	Shampoo	Moisturizing, UV-protecting, long-lasting skin protection	[161]
*Laminaria japonica* powder	Solid soap	Moisturizing, smoothing,	[162]
*Ascophyllum nodosum*, *Fucus vesiculosus* extracts	Serum	Energizing, antioxidants	[163]

## Data Availability

Data are contained within this article.
